# Multisensory integration in rodent tactile but not visual thalamus

**DOI:** 10.1038/s41598-018-33815-y

**Published:** 2018-10-24

**Authors:** Malte Bieler, Xiaxia Xu, Annette Marquardt, Ileana L. Hanganu-Opatz

**Affiliations:** 1 Developmental Neurophysiology, Institute of Neuroanatomy, University Medical Center Hamburg-Eppendorf, 20251 Hamburg, Germany; 2 Present Address: Laboratory for Neural Computation, Department of Physiology, University of Oslo, 0372 Oslo, Norway

## Abstract

Behavioural performance requires a coherent perception of environmental features that address multiple senses. These diverse sensory inputs are integrated in primary sensory cortices, yet it is still largely unknown whether their convergence occurs even earlier along the sensory tract. Here we investigate the role of putatively modality-specific first-order (FO) thalamic nuclei (ventral posteromedial nucleus (VPM), dorsal lateral geniculate nucleus (dLGN)) and their interactions with primary sensory cortices (S1, V1) for multisensory integration in pigmented rats *in vivo*. We show that bimodal stimulation (i.e. simultaneous light flash and whisker deflection) enhances sensory evoked activity in VPM, but not dLGN. Moreover, cross-modal stimuli reset the phase of thalamic network oscillations and strengthen the coupling efficiency between VPM and S1, but not between dLGN and V1. Finally, the information flow from VPM to S1 is enhanced. Thus, FO tactile, but not visual, thalamus processes and relays sensory inputs from multiple senses, revealing a functional difference between sensory thalamic nuclei during multisensory integration.

## Introduction

Integration of diverse sensory inputs into a unified percept enables optimal interaction with the environment by decreasing reaction times and amplifying behaviourally relevant stimuli^1–4^. The combinatorial processing of information takes place at multiple points along the sensory tract. These include not only secondary and association areas, but also the putatively unisensory primary cortices^5–7^. Modulation of network oscillations in their power and phase is a powerful mechanism of multisensory integration^7,8^. This mechanism is thought to rely upon direct axonal projections that have been documented between primary sensory cortices in rodents^7,9–15^. By these means, neurons in primary sensory cortices carry information from multiple senses either by modulating their firing rate (i.e. rate coding) or by sharpening the coincidence of spiking (i.e. temporal coding)^16–18^.

However, recent results showed that integration of multiple senses is not restricted to neocortical areas, but might take place earlier along the sensory tract, i.e. at the thalamic level. Selective blockade of electrical activity in the primary sensory cortices did not abolish, but rather enhanced the magnitude of bimodal interactions^7^. The bottom-up subcortical contribution to integrative processing is supported by the visual modulation of auditory thalamic responses in medial geniculate body (MGB)^19^ and the convergence of visual and tactile responses in the VPM^20^. Both FO thalamic nuclei send direct subcortical information to the cortex, in contrast to higher-order (HO) thalamic nuclei that provide a trans-thalamic relay from one part of the cortex to another^21^. Detection sensitivity for visual stimuli has been shown to relate to the coupling strength between FO thalamus and cortical regions when auditory stimuli are presented concurrently^3^. The anatomical connectivity between thalamic nuclei and primary sensory areas has been recently characterised^22,23^, yet the functional mechanisms of their interactions during cross-modal stimulation are still poorly understood.

Here, we aim at elucidating the contribution of FO thalamic nuclei to the processing of visual-tactile information. To this end, we combine simultaneous multi-site extracellular recordings from S1 and V1 as well as from VPM and dLGN with retrograde tracing of axonal connectivity within thalamo-thalamic and thalamo-cortical circuits of pigmented Brown Norway rats. We provide evidence that, in contrast to dLGN, cross-modal inputs consistently and significantly modulate network oscillations and neuronal firing patterns in VPM.

## Results

### Evoked potentials in VPM and dLGN as a result of unimodal versus bimodal visual-tactile stimulation

Throughout the study we use the following terms as defined here: *unimodal* describes a modality-specific stimulus of one single sensory modality; *cross-modal* describes a modality non-specific stimulus; *bimodal* describes two co-occurring stimuli from different sensory modalities, e.g. simultaneous visual and tactile stimulation; *multisensory integration* describes a process involving multisensory processing that leads to a different neural response after bimodal when compared to unimodal stimulation (i.e. a statistically significant difference in the rate and/or temporal pattern of spiking activity at the level of single neurons, or a statistically significant difference of neural activity at subthreshold/local field potential (LFP) level).

**Figure 1:**
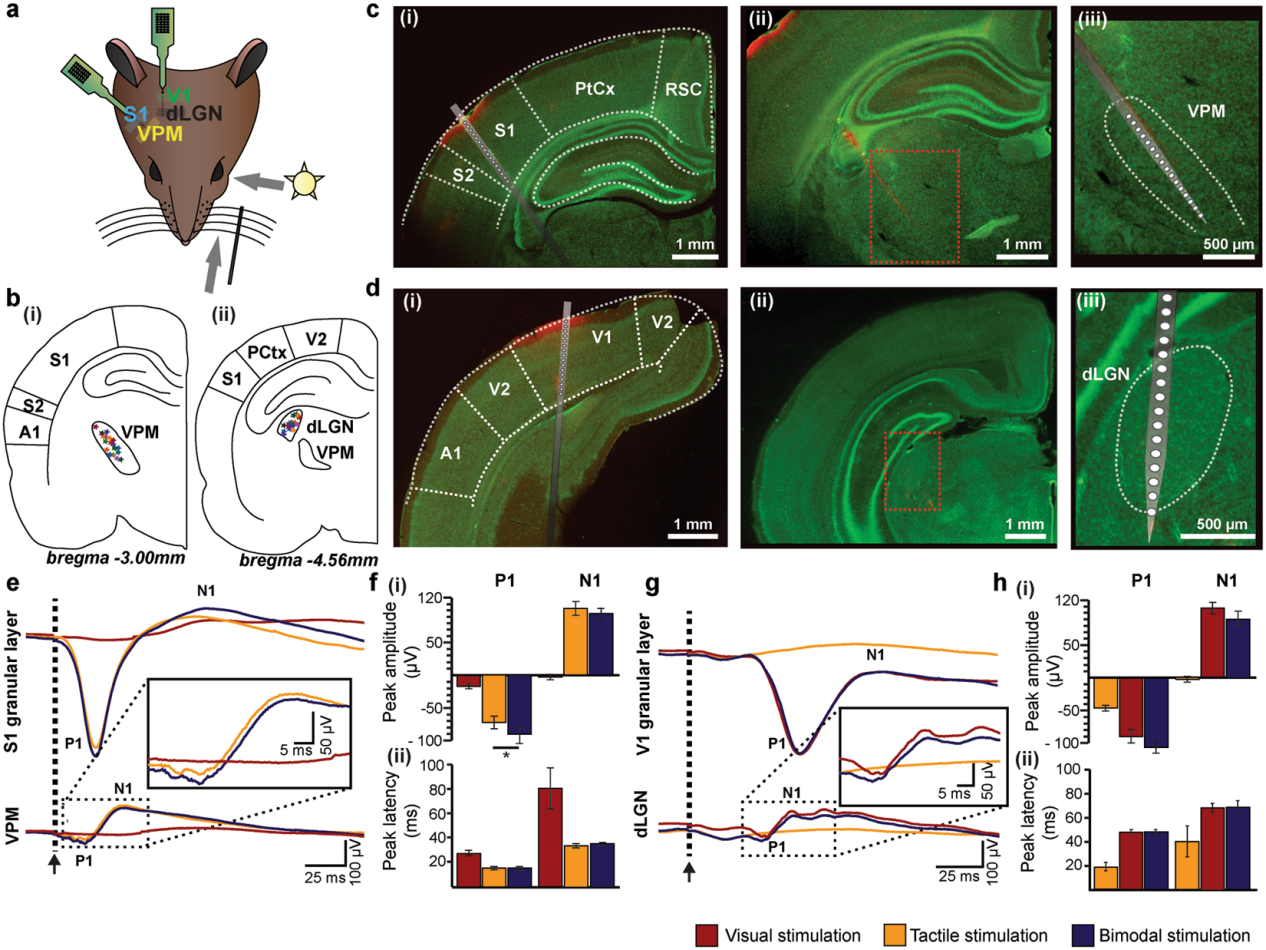
Uni- versus bimodal evoked responses in primary sensory cortices and corresponding first-order thalamic nuclei. **(a)** Schematic drawing displaying the sensory stimulation via light flashes and whisker deflections as well as the position of recording electrodes in S1, VPM, V1, and dLGN of a Brown Norway rat. **(b)** (i) Schematic drawing displaying the location of all recording sites in VPM (n = 18) that were used for the analysis of the LFP and SUA. (ii) Same as (i) for dLGN. **(c)** (i) Nissl stained coronal section displaying the location of the custom-made DiI-labelled multi-site recording electrode in the S1 of a Brown Norway rat. Note that the silicon probe is inserted angularly to target both S1 and VPM. (ii) Same as (i) for VPM. (iii) Nissl stained coronal section displaying the position of recording sites in the VPM in (ii) when displayed at higher magnification. **(d)** Same as (c) for V1 and dLGN. **(e)** Positive (P1) and negative (N1) peaks of EPs averaged across animals after visual (red), tactile (orange) and bimodal visual-tactile (blue) stimulation in granular layers of S1 (top) and VPM (bottom). Inset displays the EP in VPM at larger magnification. Black arrow marks the stimulation. **(f)** (i) Bar diagrams displaying the maximum amplitude of P1 and N1 across animals after visual (red), tactile (orange) and bimodal visual-tactile (blue) stimulation in VPM. (ii) Bar diagrams displaying the latency of the maximum amplitude of P1 (left) and N1 (right) after tactile (orange) and bimodal visual-tactile (blue) stimulation in VPM. **(g)** Same as (**e**) for granular layers of V1 and dLGN. **(h)** Same as (**f**) for dLGN. A1 = primary auditory cortex, PtCx = parietal cortex, RSC = retrosplenial cortex. (^*^p < 0.05, ^**^p < 0.01, ^***^p < 0.001; paired sample t-test). The schematic drawing in (**a**) was obtained and modified from Bieler *et al*^80^. *Visual-tactile processing in primary somatosensory cortex emerges before cross-modal experience*. Synapse. 2017 Jun;71(6). John Wiley & Sons, Inc., Hoboken, NJ, USA.

To get first insights into the convergence of visual and tactile information in FO thalamus, we assessed the impact of unimodal and bimodal stimulation on the LFP in VPM and dLGN when simultaneously recorded with the primary sensory cortices S1 and V1 of postnatal day (P) 19–23 Brown Norway rats (n = 18) (Fig. 1a–d). All recordings were conducted under urethane anaesthesia to avoid the impact of spontaneous whisking and varying alert states on the feedforward mechanisms of multisensory processing^24^. The good visual acuity of pigmented Brown Norway rats compared to albino rats^25^ makes them well-suited for testing visual-tactile processing. Thalamic and cortical regions were targeted according to stereotaxic coordinates^26^ that were confirmed post-mortem by reconstructions of the electrode tracks confined to the area of interest (Fig. 1b–d) as well as according to electrophysiological landmarks (evoked potentials (EPs) in S1, V1, VPM, dLGN, and layer 4 reversal in S1 and V1).

In the absence of sensory stimulation, neural activity in VPM and dLGN was characterized by network oscillations in delta (1–4 Hz) to beta-low gamma (12–45 Hz) frequency range. These spontaneous rhythms in VPM and dLGN had a similar temporal structure and low amplitude when compared with the simultaneously recorded activity of S1 and V1 (Supplementary Fig. S1). Their power peaked in the delta-theta band (Supplementary Fig. 1, Supplementary Table S1). Even in the absence of stimuli, multi-unit activity (MUA) discharge of thalamic neurons was prominent (VPM: 7.94 ± 2.78 Hz; dLGN: 4.47 ± 1.97 Hz) (Supplementary Fig. S1).

Tactile stimulation led to evoked responses with fast peaks (P1) followed by slower components (N1) in VPM and S1 (Fig. 1e). Previous investigations showed that bimodal stimulation enhances the amplitude of EPs in S1^7^. Similarly, the amplitude of P1 of VPM EPs was significantly (paired sample t-test, n = 18, t = −2.62, p < 0.018) enhanced after bimodal visual-tactile stimulation (tactile: −73.08 ± 9.51 µV, bimodal: −91.37 ± 13.97 µV), whereas the amplitude of N1 remained unaffected (paired sample t-test, n = 18, t = −1.985, p = 0.063, tactile: 107.73 ± 10.97 µV, bimodal: 94.93 ± 8.87 µV) (Fig. 1fi, Table 1). The multisensory enhancement of the P1 response in VPM was not supra-additive, as the arithmetic sum of the P1 amplitude after visual and after tactile stimulation was not significantly larger when compared to bimodal visual-tactile stimulation (paired sample t-test, n = 18, t = 0.058, p = 0.96, arithmetic sum visual and tactile: −91.83 ± 13.13 µV, bimodal: −91.37 ± 13.97 µV). Bimodal stimulation did not decrease the latency of the P1 peak in VPM (paired sample t-test, n = 18, t = 0.59, p = 0.633, tactile: 15.41 ± 1.4 ms, bimodal: 15.66 ± 1.38 ms) (Fig. 1fii, Table 1). Thus, visual stimulation modulated the tactile evoked responses in VPM. This effect was variable as revealed by visual inspection of averaged EP traces across trials of individual animals. Overall, 10 animals showed an increase in the VPM P1 peak after bimodal visual-tactile stimulation when compared to unimodal tactile stimulation, 6 animals a decrease and 2 animals displayed no change (as compared to S1 where 12 animals displayed an increase and 6 animals a decrease in the amplitude of the EP after bimodal visual-tactile stimulation). The source of these variable responses is unknown, but it might represent the variable processing along sensory tracts^27,28^.

**Table 1:** Peak amplitude and latency of EPs in first-order thalamus and primary sensory cortex after unimodal and bimodal stimulation.

	P1 amplitude (µV)			P1 latency (ms)			N1 amplitude (µV)			N1 latency (ms)		
	Visual	Tactile	Bimodal	Visual	Tactile	Bimodal	Visual	Tactile	Bimodal	Visual	Tactile	Bimodal
VPM	−18.75 ± 3.62	−73.08 ± 9.51^*^	−91.37 ± 13.97	27.41 ± 2.18	15.41 ± 1.40	15.66 ± 1.38	−2.25 ± 3.92	107.735 ± 10.97	94.93 ± 8.87	82.80 ± 17.20	33.79 ± 1.69	35.79 ± 0.52
S1	−23.44 ± 5.14	−528.11 ± 48.99^*^	−550.61 ± 52.60	64.13 ± 15.91	26.69 ± 7.27	26,67 ± 7.38	49.78 ± 5.28	77.22 ± 9.45	94.00 ± 13.06	107.18 ± 23.78	77.62 ± 20.38	79.88 ± 19.34
dLGN	−91.21 ± 11.21	−45.57 ± 4.34	−105.41 ± 10.09	46.63 ± 2.20	18.43 ± 3.52	46.99 ± 2.29	106.325 ± 8.90	−3.04 ± 4.45	91.26 ± 10.61	67.47 ± 3.73	40.75 ± 11.49	68.25 ± 4.10
V1	−422.83 ± 39.57	−33.22 ± 4.00	−419.22 ± 42.09	60.07 ± 5.57	33.93 ± 9.17	60.79 ± 5.92	88.28 ± 16.56	21.94 ± 4.34	82.83 ± 15.27	96.74 ± 7.55	78.13 ± 12.91	97.28 ± 8,16

Values are mean ± SEM for evoked responses in VPM, S1, dLGN and V1 after unimodal and bimodal stimulation. *p < 0.05, paired sample t-test.

The prominent changes caused by bimodal stimulation in VPM seemed to be structure-specific, since visual-tactile stimulation did not similarly affect the EPs in dLGN along the visual tract (Fig. 1g,h, Table 1).

Thus, co-occurring visual stimuli facilitate the tactile evoked responses in VPM, yet tactile modulation of sensory evoked responses was absent in dLGN and V1.

### Induced oscillatory activity in the VPM and dLGN as a result of unimodal versus bimodal visual-tactile stimulation

In addition to the spontaneous and stimulus-evoked network activity, stimulus-induced oscillations have been detected (Supplementary Fig. S2). In contrast to the stimulus-evoked activity, stimulus-induced network oscillations are causally related but not phase-locked to the sensory stimuli. Previous studies showed that in the superior colliculus (SC) and primary sensory cortices these patterns of oscillatory activity are profoundly modulated by bimodal stimulation^7,17,29–31^. To test whether visual-tactile stimulation modulates network activity in the corresponding FO thalamic nuclei, we assessed the temporal dynamics of stimulus-induced oscillations in different frequency bands. First, we analysed individual frequency spectra corresponding to a large number of unimodal stimulation trials. In VPM, shortly after the stimulus-evoked response (i.e. 50–150 ms post-stimulus), the amplitude of stimulus-induced oscillations significantly increased particularly in the 1–4 Hz, 4–8 Hz, and 8–12 Hz frequency bands when compared with baseline conditions (i.e. 200–300 ms pre-stimulus). In dLGN, unimodal visual stimulation led to similar modulation of network oscillations as observed after whisker deflections in VPM (Supplementary Fig. S2).

**Figure 2:**
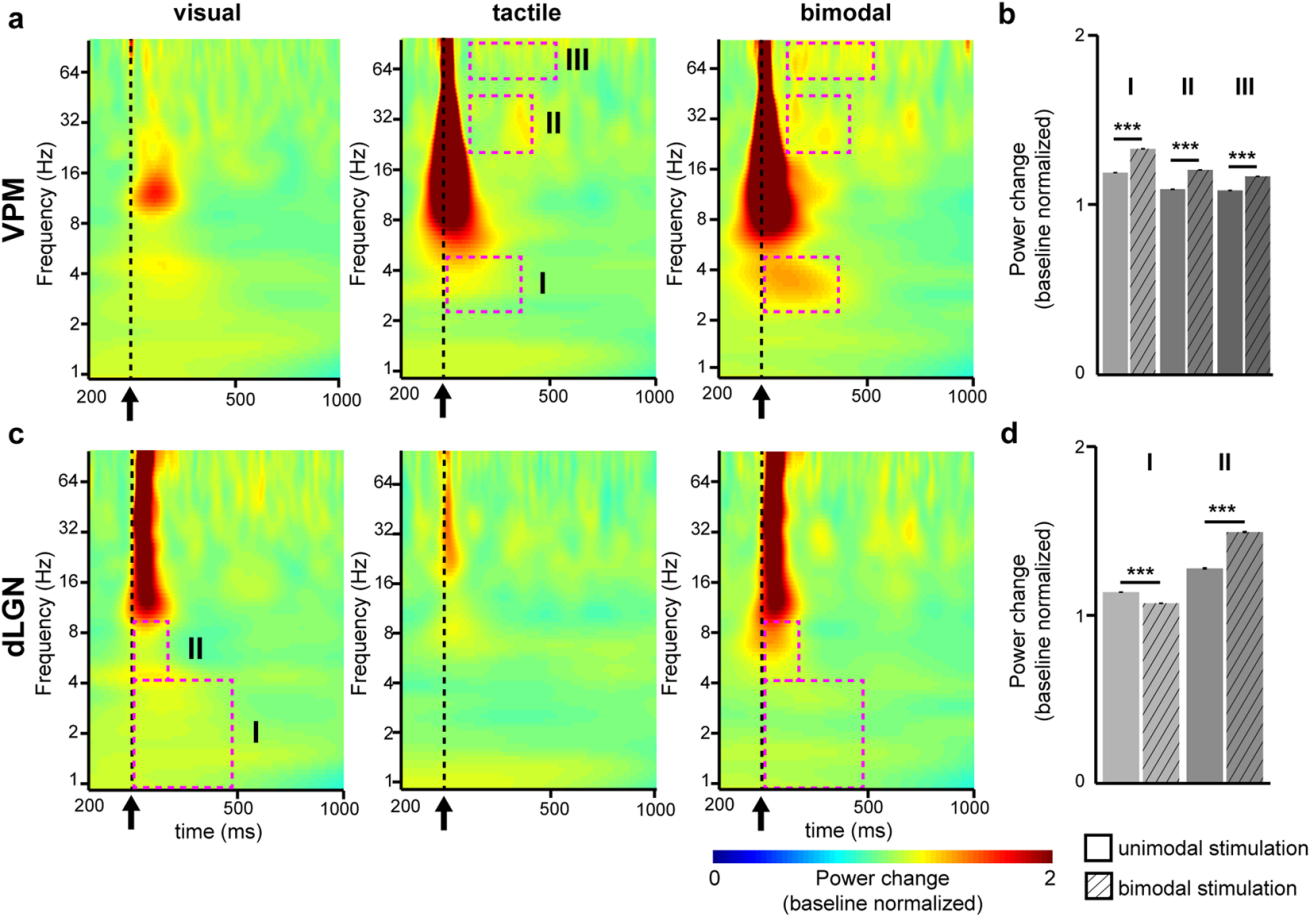
Uni- and bimodal induced responses in first-order thalamic nuclei. **(a)** Baseline normalised Morlet wavelet spectra of the LFP in VPM averaged for all rats (n = 18) 200 ms pre-stimulus and 1000 ms post-stimulus after unimodal visual (left), unimodal tactile (middle) and bimodal visual-tactile (right) stimulation. Magenta dotted boxes indicate significant time and frequency windows of power modulation (z-score <= −2.56/z-score >= 2.56) after bimodal stimulation when compared to unimodal tactile stimulation. Black arrow marks the stimulation. **(b)** Bar diagram displaying the mean power of oscillatory activity in VPM during previously defined post-stimulus time-windows normalised to the power of activity before stimulation (baseline). **(c)** Same as (**a**) for dLGN after bimodal visual-tactile stimulation when compared to unimodal visual stimulation. **(d)** Same as (b) for dLGN. (*p < 0.05, **p < 0.01, ***p < 0.001; Wilcoxon signed-rank test).

Second, we assessed the effects of bimodal visual-tactile stimulation on the induced oscillatory activity in FO thalamus. For this, we pooled the multiple-trial baseline normalised Morlet wavelet spectra across animals (n = 18) separately for the three different conditions: unimodal tactile, unimodal visual and bimodal visual-tactile stimulation. Visual or tactile stimulation led to minor modifications of the frequency distribution in VPM or dLGN, respectively (Fig. 2a,c). Unimodal stimulation (tactile for VPM and visual for dLGN) modulated the power of oscillatory activity mainly in beta-low gamma band (20–45 Hz) in VPM and in delta (1–4 Hz) and gamma band (50–128 Hz) in dLGN. The modulatory effects augmented during bimodal visual-tactile stimulation. To assess the power of oscillatory activity in VPM and dLGN after bimodal visual-tactile stimulation, we compared the baseline-normalised power range for each time-frequency point of the Morlet wavelet spectra. The time points with significant power changes after bimodal versus unimodal stimulation were clustered using a previously developed protocol^7^. In VPM, three such time-windows were identified in delta (Wilcoxon signed-rank test, n = 100035 time-frequency values, Z = −90.04, p < 0.001, tactile: 1.19 ± 0.0007, bimodal: 1.33 ± 0.0007), beta-low gamma (Wilcoxon signed-rank test, n = 7000 time-frequency values, Z = −75.50, p < 0.001, tactile: 1.09 ± 0.0006, bimodal: 1.21 ± 0.0004), and gamma frequency (Wilcoxon signed-rank test, n = 14400 time-frequency values, Z = −97.04, p < 0.001, tactile: 1.09 ± 0.0003, bimodal: 1.16 ± 0.0004) (Fig. 2a,b). In dLGN, two clustered time-windows with significant power changes in delta (Wilcoxon signed-rank test, n = 40425 time-frequency values, Z = 177.33, p < 0.001, visual: 1.12 ± 0.0001, bimodal: 1.06 ± 0.0001) and theta band (Wilcoxon signed-rank test, n = 2170 time-frequency values, Z = −42.41, p < 0.001, visual: 1.26 ± 0.003, bimodal: 1.48 ± 0.003) were detected (Fig. 2c,d). With the exception of delta power in dLGN, which decreased after visual-tactile stimulation, bimodal stimulation augmented the oscillatory power of network oscillations in FO thalamic nuclei in a frequency-specific pattern (Fig. 2b,d).

These results indicate that at the level of FO thalamus, visual-tactile stimulation modifies the power of induced network oscillations in VPM, while this effect is inconsistent in dLGN.

### Cross-modal phase reset as a mechanism of multisensory integration in first-order thalamus

**Figure 3:**
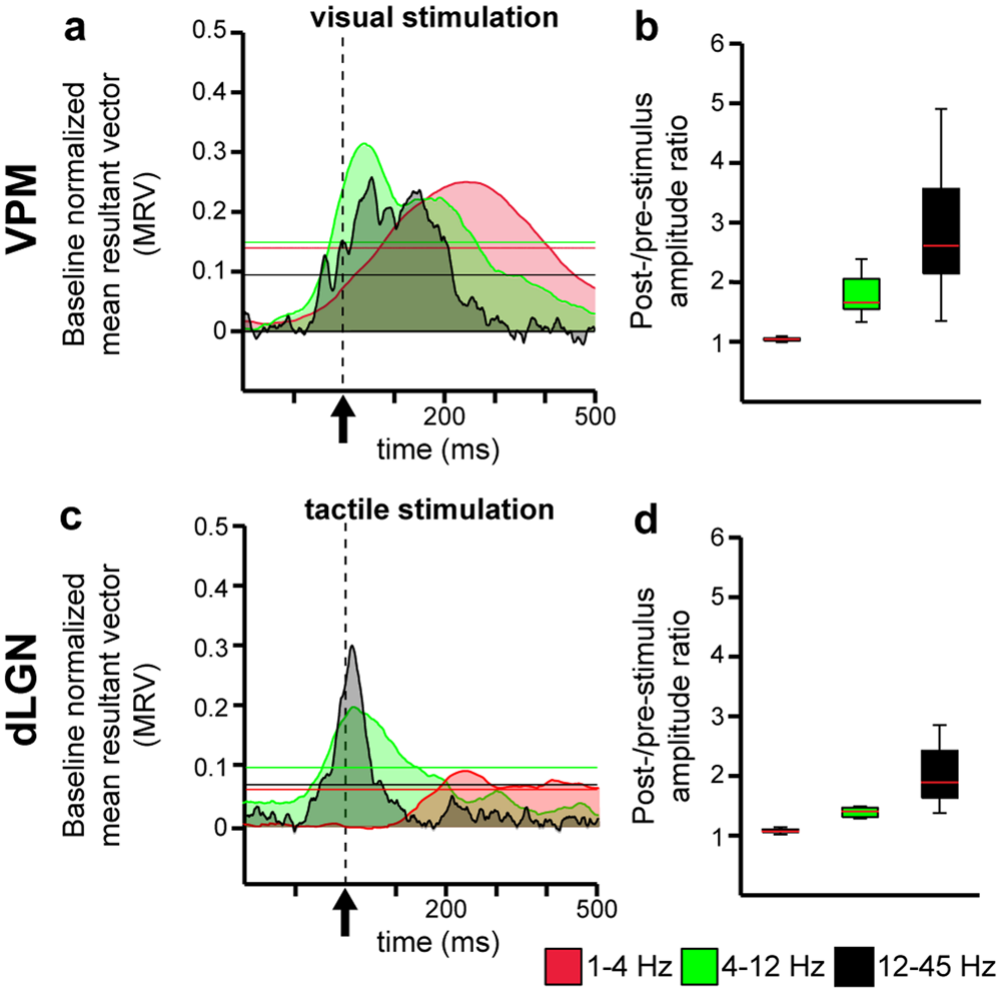
Event-related phase synchrony in first-order thalamic nuclei after visual or tactile stimulation. **(a)** Plot displaying the baseline normalised mean resultant vector (MRV) length of oscillatory phases in VPM after visual stimulation (dotted line, black arrow). The values were averaged over each time point in all 18 rats for three frequency bands, 1–4 Hz (red), 4–12 Hz (green) and 12–45 Hz (black). Note, visual stimulation caused strong and long-lasting (413 ms for 1–4 Hz, 266 ms for 4–12 Hz, 219 ms for 12–45 Hz) augmentation of phase synchrony in all frequencies compared to baseline (i.e. >99% confidence interval). **(b)** Boxplots displaying the post-/pre-stimulus power ratio for 1–4 Hz (red), 4–12 Hz (green) and 12–45 Hz (black) oscillations. **(c)** Same as (**a**) for dLGN. Note, a similar significant increase in phase synchrony (i.e. >99% confidence interval) for 4–12 Hz and 12–45 Hz as compared to VPM was found in dLGN. **(d)** Same as (**b**) for dLGN. (Horizontal coloured lines mark the border of confidence α = 0.001).

Next, we aimed to elucidate the mechanism by which the co-occurring visual-tactile stimuli modulate the evoked and induced network activity in FO thalamic nuclei. Alignment of oscillatory activity to a specific phase has been proposed to prime the neural system about forthcoming sensory stimuli, and by these means, to augment their effectiveness for multisensory perception and behavior^32^. While this cross-modal phase reset has been found ubiquitary in primary sensory cortices^7,8^, it remains unknown whether a similar mechanism exists at the level of FO thalamus. Therefore, we compared the phase synchrony of spontaneous oscillations in VPM and dLGN during a large number of cross-modal stimulation trials (visual stimulation when recording in VPM or tactile stimulation when recording in dLGN) and calculated the mean resultant vector (MRV) length of oscillatory phases of the previously identified spontaneous delta (1–4 Hz), theta (4–12 Hz) and beta-low gamma (12–45 Hz) activity in VPM and dLGN (Supplementary Fig. S1). If the oscillatory phase is the same in each trial, the MRV length will be 1, whereas if the oscillatory phase is absolutely random, the value will be 0 (Fig. 3a,c). In addition, to exclude the EP-related synchrony, we calculated the post-/pre-stimulus power ratio ([0–250 ms post-stimulus]/[200–300 ms pre-stimulus])^8^. In contrast to a true phase reset, the EP-related synchrony is accompanied by a pre- to post-stimulus power increase in the single-trial responses^33,34^. Therefore, a post-/pre-stimulus power ratio of 1 and a MRV > 0 correspond to true phase synchrony. These conditions were fulfilled only by the oscillatory activity in the 1–4 Hz range in VPM, but not dLGN (Fig. 3b,d). Thus, visual stimulation enhances the phase synchrony of oscillatory activity in VPM.

### Neuronal firing in the VPM and dLGN as a result of unimodal versus bimodal visual-tactile stimulation

**Figure 4:**
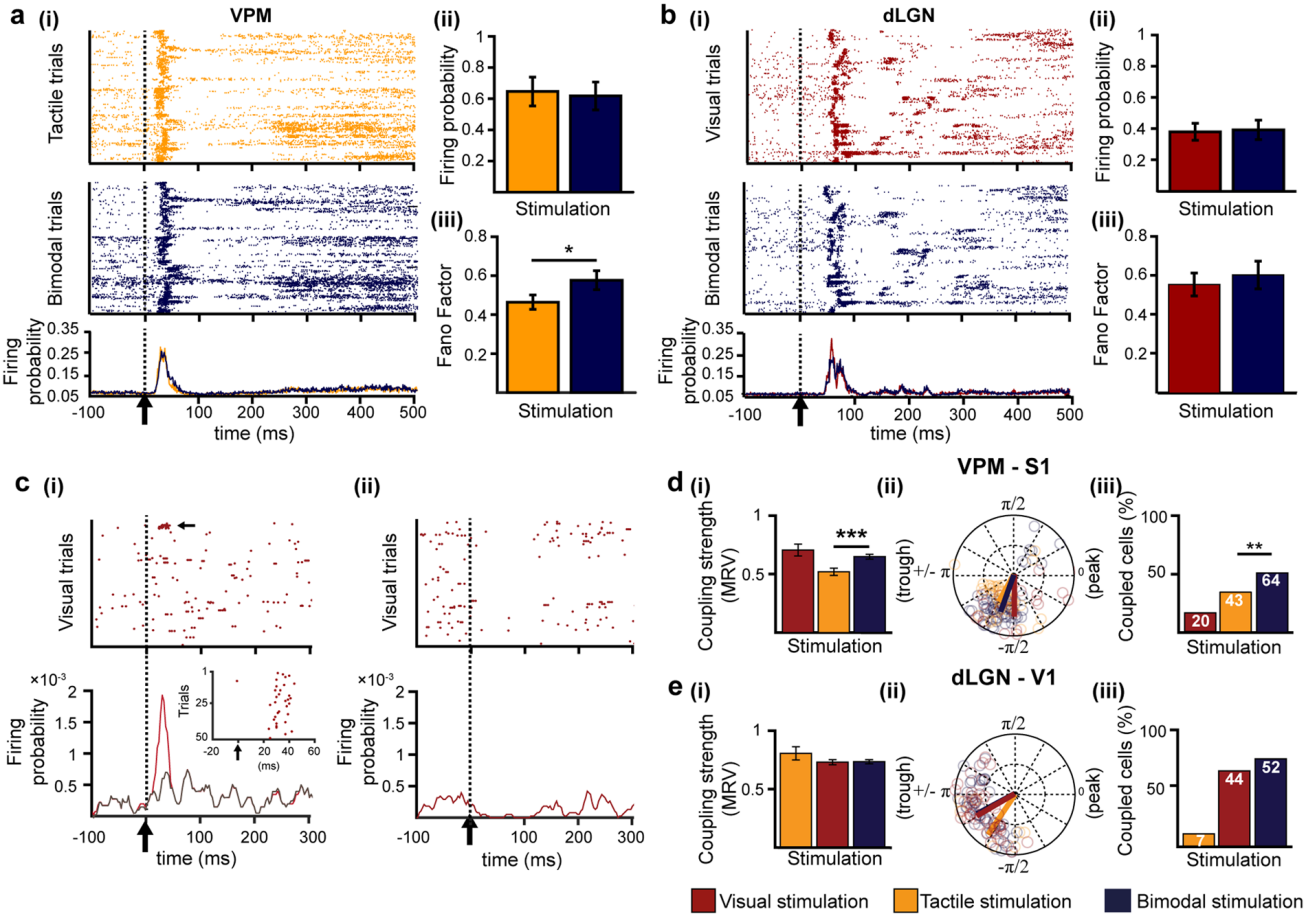
Neuronal firing in first-order thalamic nuclei after uni- and bimodal stimulation. **(a)** (i) Rasterplot depicting the firing of single cells for each trial after unimodal tactile (orange, top) and bimodal visual-tactile (blue, bottom) stimulation. (ii) Bar diagram displaying the firing probability after unimodal tactile (orange) and bimodal visual-tactile (blue) stimulation of single neurons that enhance their firing after stimulation. (iii) Bar diagram displaying the firing variability measured by the Fano factor of single neurons that enhance their firing after stimulation. **(b)** Same as (**a**) for dLGN. **(c)** (i) Rasterplot depicting the firing of single cells in VPM (12 out of 126) that showed a firing enhancement after unimodal visual stimulation (marked by dotted line and black arrow) (top) and line plot displaying the corresponding firing probability (bottom). Inset displays the firing response of the strongly responding neuron marked by black arrow above. Grey line corresponds to the firing probability after exclusion of the strong visual responsive neuron. (ii) Same as (i) for neurons in VPM that showed a depression of firing probability after visual stimulation (38 out of 126). **(d)** (i) Bar diagram displaying the MRV of VPM-S1 spike-LFP locking strength 0–80 ms after visual (red), tactile (orange) and bimodal visual-tactile (blue) stimulation. (ii) Polar plot displaying the coupling of spikes of VPM neurons to the phase of 1–4 Hz network oscillations in S1. (iii) Bar diagram displaying the fraction of significantly phase-locked neurons. White numbers indicate the total number of significantly phase-locked neurons after stimulation. **(e)** Same as (**f**) for dLGN spikes phase locked to 1–4 Hz oscillations in granular layers of V1. (*p < 0.05, **p < 0.01, ***p < 0.001; one sample t-test, z-test for two proportions).

Previous studies revealed that the information content from multiple senses is encoded in primary sensory cortices not only at population level but also by the firing of individual neurons^16,35^. For this, both rate and temporal coding strategies are used. To assess whether single thalamic neurons process multisensory information by means of rate and temporal coding, we examined single unit activity (SUA) obtained by clustering the extracellularly recorded spikes according to their waveform shape. The patterns of thalamic firing resembled the previously described spiking patterns of cortical neurons after visual, tactile and bimodal visual-tactile stimulation (Supplementary Fig. 3)^16^. Only neurons that responded to stimulation by increasing their firing rate after unimodal and bimodal stimulation were considered for further analyses. In the VPM, unimodal tactile stimulation led in a large fraction of neurons (75 out of 126) to a strong firing increase peaking at 16.64 ± 0.63 ms after the stimulus (Fig. 4a, Table 2). When compared to baseline, the firing increase in VPM was followed by a non-significant decrease and subsequently, by a long-lasting low-magnitude augmentation of firing beginning 200 ms after stimulation (Fig. 4a). A fraction of neurons in VPM (50 out of 126) responded to visual stimuli by either augmenting or depressing their firing probability (Fig. 4c, Table 2). The firing of dLGN neurons was similarly affected by unimodal visual and bimodal visual-tactile stimulation (Fig. 4b), yet only 19 out of 66 dLGN neurons changed their firing probability after tactile stimulation, and the majority of those neurons (14 out of 19) displayed a reduction in firing probability (Table 2).

**Table 2:** Firing patterns of thalamic neurons after uni- and bimodal stimulation.

		VPM	dLGN
*Enhancement*	Stimulation		
	*Tactile*	75	5
	*Visual*	12	48
	*Bimodal*	73	50
*Depression*	Stimulation		
	*Tactile*	16	14
	*Visual*	38	5
	*Bimodal*	19	6
*No change*	Stimulation		
	*Tactile*	35	47
	*Visual*	76	13
	*Bimodal*	34	10
Total		126	66

Thalamic neurons showed either an enhancement in firing probability after stimulation when compared to baseline, a depression in firing probability or no change. Change in firing probability was defined as +/− five times the standard deviation of the baseline firing probability.

Though the majority of clustered units in VPM (75 out of 126) enhanced their firing shortly after stimulation, there was no statistically significant difference in the augmentation of firing probability between unimodal and bimodal stimulation (Fig. 4aii). However, the neuronal firing after bimodal stimulation was more variable, since the Fano factor (FF), which captures the response variability of single neurons, increased when compared to unimodal tactile stimulation (one sample t-test, n = 59 units, t = 2.02, p = 0.04, tactile: 0.45 ± 0.04, bimodal: 0.57 ± 0.05) (Fig. 4aiii). Given that a similar amount of VPM neurons reponded to bimodal and unimodal stimulation, the increase in variance can most likely be attributed to a few individual neurons that increased their firing variability rather than to a population effect. Bimodal stimulation neither affected the firing probability (one sample t-test, n = 48 units, t = 0.44, p = 0.66, visual: 0.39 ± 0.06, bimodal: 0.41 ± 0.06) nor the firing variability of neurons in dLGN (one sample t-test, n = 48 units, t = 0.74, p = 0.46, visual: 0.55 ± 0.06, bimodal: 0.61 ± 0.07) (Fig. 4bii,iii).

To decide whether thalamic neurons process multisensory information using a temporal code, we assessed the impact of visual-tactile stimulation on the timing of neuronal firing in FO thalamic nuclei to network oscillations in primary sensory cortices. The analysis was performed during the first 80 ms after sensory stimulation, the time window with the strongest firing response. The spike-LFP coupling analysis revealed that VPM neurons increase their locking strength to delta oscillations in granular (G) layers of S1 after bimodal visual-tactile stimulation when compared to unimodal tactile stimulation (circ_cm test, n = 43 units tactile, n = 64 units bimodal, p = 0.0001, tactile: 0.52 ± 0.03, bimodal: 0.65 ± 0.02) (Fig. 4di,ii). The enhancement of VPM-S1 spike-LFP coupling strength was accompanied by a significant increase in the number of VPM neurons significantly phase-locked to delta oscillations in G layers of S1 after bimodal visual-tactile stimulation when compared to unimodal tactile stimulation (two proportion z-test, n = 126 units, Z = −2.68, p = 0.007, tactile: 43 out of 126 neurons, bimodal: 64 out of 126 neurons) (Fig. 4diii). This was not the case for the spike-LFP coupling in the dLGN-V1 circuit (two proportion z-test, n = 66 units, Z = −1.56, p = 0.12, visual: 44 out of 66 neurons, bimodal: 52 out of 66 neurons) (Fig. 4e). Few VPM neurons (20 out of 126 neurons) timed their firing to S1 network oscillations after unimodal visual stimulation as well, though their locking strength was not augmented when compared to bimodal stimulation (circ_cm test, n = 20 units visual, n = 64 units bimodal, p = 0.60, visual: 0.71 ± 0.05, bimodal: 0.65 ± 0.02) (Fig. 4d).

Finally, we tested whether VPM neurons apply a dual rate and temporal coding strategy to process and relay multisensory information. We found that the majority of VPM neurons that are phase-locked to S1 delta oscillations also showed an enhancement in firing probability after bimodal visual-tactile stimulation (tactile: 37 out of 43 phase-locked neurons showed an increase in firing probability, bimodal: 55 out of 64 phase-locked neurons showed an increase in firing probability). In contrast, visual responsive neurons in VPM that were phase-locked to delta oscillations in G layers of S1 were less likely to display an increase in their firing probability (4 neurons out of 12). Thus, unimodal as well as multisensory neurons in VPM display a dual rate and temporal coding mechanism, which was absent for visually responsive neurons in VPM.

Taken together, these results indicate that neurons in VPM largely enhance their firing probability after tactile and bimodal stimulation. Few VPM neurons respond to visual stimulation. Overall, VPM neurons process multisensory stimulation by increasing their spiking variability and timing their firing output to ongoing network oscillations in S1 when compared to unimodal tactile processing. These effects were absent in dLGN neurons.

### Thalamo-cortical coupling as a result of unimodal versus bimodal visual-tactile stimulation

The data above indicate that the FO thalamic nuclei, in particular VPM, are sites of multisensory integration. Relaying visual-tactile information from FO thalamus to primary sensory cortices by timing the firing output of single thalamic neurons to cortical network oscillations is an efficient neuronal coding mechanism. Next, we investigated in more detail how visual-tactile stimuli are transferred from the thalamic to cortical level.

**Figure 5:**
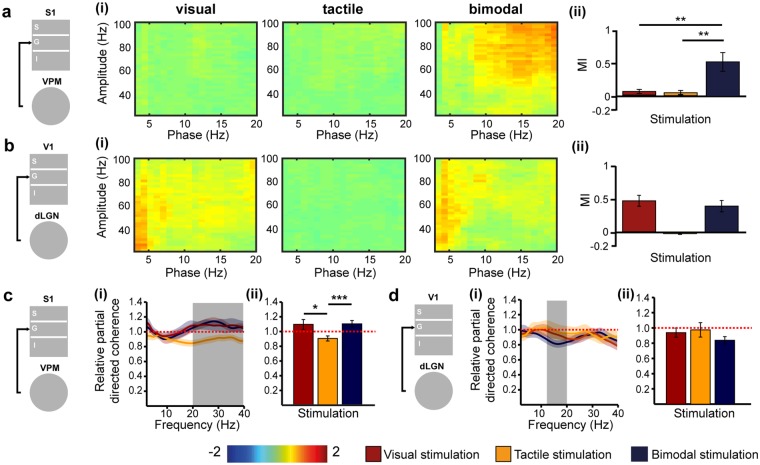
Thalamo-cortical phase-amplitude cross-frequency coupling and directed interactions after uni- and bimodal stimulation. **(a)** Schematic diagram of assessed coupling. (i) Heat maps displaying the relative (post-stimulation – pre-stimulation) phase amplitude cross-frequency coupling (CFC) between VPM and G layers of S1 after visual, tactile and bimodal stimulation. (ii) Bar diagram displaying the averaged relative modulation index (MI) for visual (red), tactile (orange) and bimodal visual-tactile (blue) stimulation. **(b)** Same as (**a**) for the coupling between dLGN and G layers of V1. **(c)** Schematic diagram of assessed directed interactions. (i) Plot displaying the relative partial directed coherence change (post-stimulus/pre-stimulus) 100–600 ms after unimodal visual (red), unimodal tactile (orange) and bimodal visual-tactile (blue) stimulation when assessed for 2–40 Hz oscillations in VPM and G layers of S1. (ii) Bar diagram displaying the relative partial directed coherence change (post-stimulus/pre-stimulus) 100–600 ms after unimodal visual (red), unimodal tactile (orange) and bimodal visual-tactile (blue) stimulation when assessed for the frequency range marked in (i) by the grey box. **(d)** Same as (**c**) for the information flow between dLGN and G layers of V1. Red dotted lines mark the border of an enhanced information flow (>1) or decreased information flow (<1.0). (*p < 0.05, **p < 0.01, ***p < 0.001; paired sample t-test).

Phase-amplitude cross-frequency coupling (CFC) has been identified as a powerful mechanism of regulating multi-scale networks and transferring information between areas after sensory events^36^. This mechanism has received little attention in multisensory research despite its critical role in memory and executive processing^37–39^. In a first step, we assessed the thalamo-cortical phase-amplitude CFC along the tactile processing stream between VPM and G layers of S1. We calculated the relative change [(post-stimulus) – (pre-stimulus)] of the modulation index (MI), which captures the CFC induced by stimulation. Bimodal stimulation significantly augmented the coupling of theta-beta phase in VPM to the amplitude of gamma oscillations in the G layers of S1 when compared to unimodal tactile stimulation (one-tailed paired t-test, n = 17, t = 3.21, p = 0.002, tactile: 0.05 ± 0.04, bimodal: 0.62 ± 0.18) (Fig. 5a). Notably, the amplitude of gamma oscillations (30–100 Hz) in G layers of S1 significantly increased after both unimodal (one-tailed paired t-test, n = 17, t = −5.08, p < 0.001, pre-stimulation: 751.54 ± 102.95 µV^2^, post-stimulation: 1180.38 ± 154.57 µV^2^) and bimodal stimulation (one-tailed paired t-test, n = 17, t = −3.58, p = 0.001, pre-stimulation: 778.87 ± 116.05 µV^2^, post-stimulation: 1151.79 ± 160.77 µV^2^). Thus, despite the presence of cortical gamma oscillations induced by unimodal tactile and bimodal visual-tactile stimulation, the coupling between VPM beta and S1 gamma oscillations is only enhanced after bimodal stimulation. In a second step, we assessed the thalamo-cortical phase-amplitude CFC along the visual processing stream. The MI was not significantly modulated after bimodal visual-tactile stimulation when compared to unimodal visual stimulation for CFC between dLGN and G layers of V1 (one-tailed paired t-test, n = 17, t = −0.69, p = 0.25, visual: 0.56 ± 0.10, bimodal: 0.47 ± 0.11) (Fig. 5b).

Thus, a change in coupling strength between VPM and G layers of S1 was only detected after bimodal visual-tactile stimulation despite the presence of induced cortical gamma oscillations by uni- and bimodal stimulation. The MI was similar after uni- and bimodal stimulation in dLGN. Thus, phase-amplitude CFC is a mechanism by which bimodal information is processed and conveyed between VPM and S1.

### Directionality of information flow within thalamo-cortical circuits as a result of unimodal versus bimodal visual-tactile stimulation

The FO thalamic nuclei strongly project to G layers of primary sensory cortices^40^. By these means, modality-specific sensory information is reliably relayed from the periphery to the cortex. To decide whether co-occurring visual and tactile stimuli impact the thalamo-cortical information flow, we analysed the generalised partial directed coherence (gPDC), a measure that reflects the directionality of network interactions in different frequency bands^41^. First, we calculated the gPDC values for interactions between VPM and G layers of S1 after unimodal tactile and bimodal visual-tactile stimulation, normalised to the baseline condition (i.e pre-stimulus) (Fig. 5c). Values of relative gPDC >1 mirror an increase in the flow of sensory information between the investigated brain areas, whereas values <1 correspond to a decrease of directed interactions. Shortly (100–600 ms) after stimulus, the relative gPDC significantly increased in the 20–40 Hz frequency range when visual and tactile stimuli were presented concurrently (paired sample t-test, n = 17, t = 3.63, p = 0.001, tactile: 0.89 ± 0.04, bimodal: 1.09 ± 0.05). An augmented flow of information was not present along the visual tract, the directionality of interactions between dLGN and G layers of V1 being similar for unimodal and bimodal conditions (one-tailed paired t-test, n = 17, t = −1.24, p = 0.12, visual: 0.96 ± 0.06, bimodal: 0.86 ± 0.05) (Fig. 5d). In line with the analyses of thalamo-cortical coupling, these results indicate that bimodal visual-tactile stimulation modulates the information flow between VPM and S1 by augmenting the thalamic drive to the primary sensory cortex. In contrast, the information flow between dLGN and V1 is not modulated in a similar way.

### Anatomical substrate of visual-tactile interactions in FO thalamic nuclei

**Figure 6:**
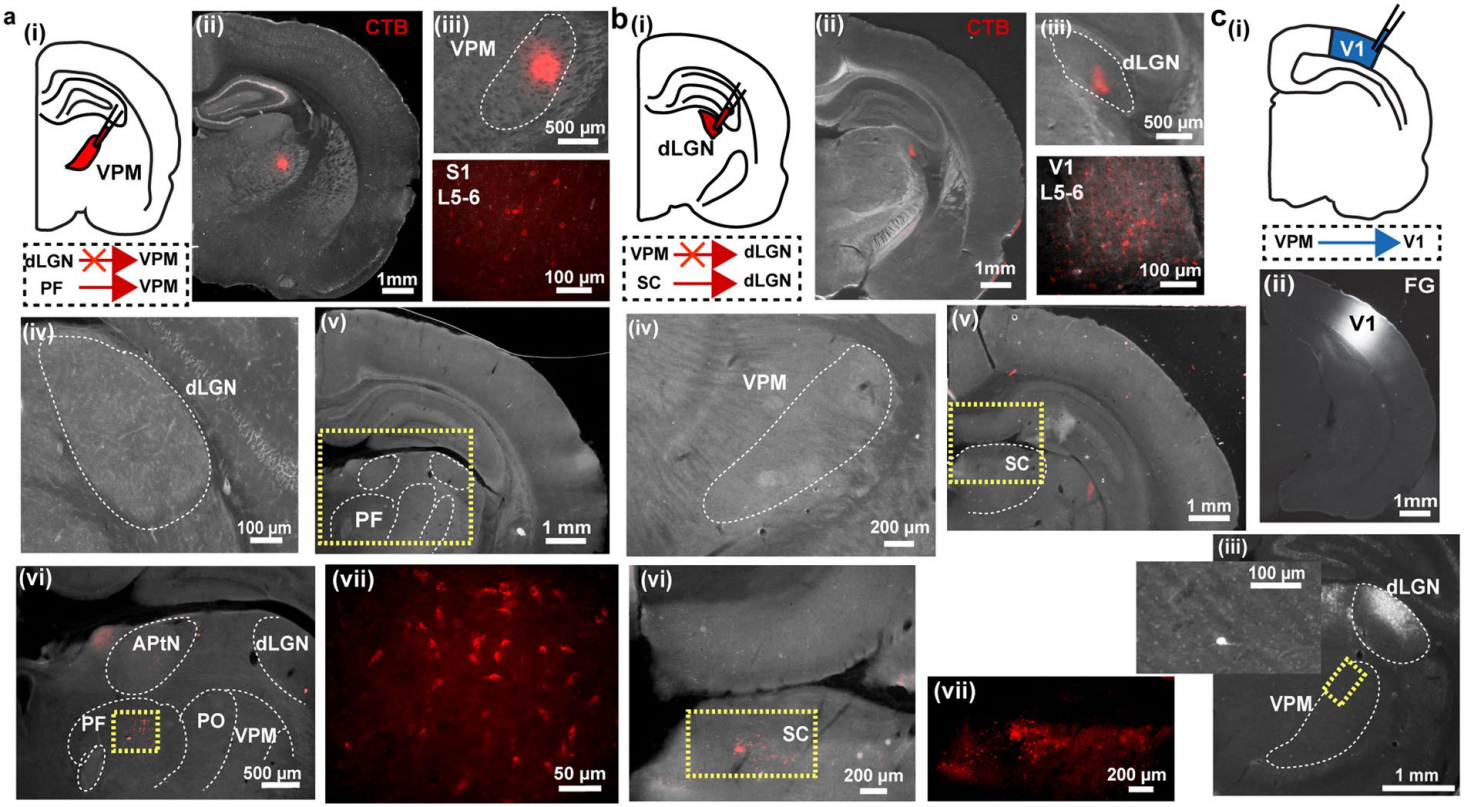
Thalamo-thalamic and thalamo-cortical connectivity revealed by retrograde tracing. **(a)** Assessment of projections from dLGN to VPM. (i) Schematic diagram displaying the site of CTB injection into VPM. (ii) Photograph displaying the CTB injection confined to VPM. (iii) Larger magnification of the CTB injection in VPM and backlabelled neurons in infragranular layers of S1. (iv) Photograph illustrating the absence of dLGN neurons projecting to VPM. (v) Photograph displaying the backlabelled neurons in the intralaminar thalamus (yellow dotted box) after CTB injections into VPM. (vi) Photograph of the intralaminar thalamus at larger magnification of the area outlined by the dotted yellow box in (v) showing backlabelled neurons in the parafascicular nucleus (PF, yellow dotted box) after CTB injections into VPM. (vii) Larger magnification of the area outlined by the dotted yellow box in (vi) showing backlabelled neurons in PF. **(b)** Same as (**a**) for CTB injection into dLGN and backlabelled cells in superficial layers of the SC. **(c)** Assessment of projections from VPM to V1. (i) Schematic diagram displaying the site of FG injection into V1. (ii) Photograph displaying the FG injection confined to V1. (iii) Photograph of dLGN and VPM with a retrogradely labelled neuron in VPM. The inset shows the neuron at larger magnification in the area outlined by the dotted yellow box. APtN = anterior pretectal thalamic nucleus, PO = posterior nucleus.

Direct axonal connectivity between S1 and V1 has been proposed as substrate of functional interactions accounting for multisensory integration at the level of primary sensory cortices^7,13,16^. The presence of similar processing mechanisms at the level of FO thalamus raises the question whether thalamo-thalamic connectivity may account for multisensory integration observed in FO nuclei or whether visual-tactile information is integrated at even earlier stages such as the midbrain or brainstem. First, we assessed the direct connectivity between VPM and dLGN by anatomical tracing. Small amounts of cholera toxin subunit B (CTB) were injected either into the VPM or dLGN by iontophoresis (VPM: n = 3, dLGN: n = 4). The procedure confined the tracer to the area of interest without reflux to neighboring areas (Fig. 6aii,iii,bii,iii). CTB injections did not reveal direct projections between VPM and dLGN. Retrogradely stained neurons were neither detected in dLGN when VPM was targeted (Fig. 6aiv) nor in VPM when CTB was confined to dLGN (Fig. 6biv). To elucidate the potential source of visual inputs to VPM and the absent tactile responses in dLGN, we further analysed retrogradely labelled cell bodies in upstream targets. We found that CTB injections into VPM revealed backlabelled cell bodies particularly in the parafiscicular nucleus (PF) of the intralaminar thalamus (Fig. 6av–vii). The PF nucleus shares anatomical connections with other brain areas, such as the SC, brainstem and basal ganglia^42,43^. The dLGN received strong projections from the superficial layers of the SC (Fig. 6bv–vii).

Second, we investigated whether VPM projects not only to S1 but also to the V1, thereby relaying multisensory information from VPM to other primary sensory cortices^7^. For this, small amounts of the retrograde tracer fluorogold (FG) were injected into V1 and covered the supragranular (S), G, and infragranular (I) layers (Fig. 6c). FG injected into V1 stained few neurons in VPM. These results confirm the previously reported sparse feedforward VPM-V1 connections^22^.

Thus, these data suggest that the putatively unimodal tactile nucleus of the thalamus receives (multi-) sensory information from other thalamic nuclei, which is then further processed and sent to putatively unimodal primary somatosensory and visual cortices, bypassing the dLGN.

## Discussion

The recently identified convergence of sensory information in primary sensory cortices raises the question of whether inputs from different senses are integrated even earlier along the sensory tracts. The present study aims to elucidate whether FO thalamic nuclei, which have been identified as major relay stations between the periphery and cortex, are involved in visual-tactile processing. By combining multi-site electrophysiology *in vivo* with anatomical tracing we demonstrate the following: (1) visual stimuli consistently impact tactile processing in VPM, but modulation of dLGN activity by tactile stimulation is inconsistent; (2) co-occurrence of visual and tactile stimuli leads to an enhancement of evoked responses and network oscillations in VPM by a cross-modal phase reset; (3) co-occurring visual-tactile stimuli enhance the coupling and directed interactions along the VPM-S1 but not the dLGN-V1 processing stream; (4) in contrast to reciprocal connections between primary sensory cortices, the FO thalamic nuclei, VPM and dLGN, are not linked via monosynaptic projections and VPM might receive retinal inputs via the intralaminar thalamus (Table 3).

**Table 3:** Bimodal effects and their underlying mechanisms in the tactile and visual thalamo-cortical processing stream.

		VPM	dLGN
*Thalamic effects*	*Evoked potentials*	**↑** P1 amplitude	—
	*Induced oscillations*	**↑** delta, beta, gamma	*inconsistent*
	*Neuronal firing*	**↑** firing variability	—
	*Phase reset*	**↑** delta	—
*Thalamo-cortical effects*	*Spike-LFP coupling*	**↑** VPM spikes – S1 delta	—
	*Phase-amplitude CFC*	**↑** beta-gamma, VPM – S1 granular	—
	*Directionality*	**↑** beta-gamma, VPM – S1 granular	—
*Anatomy*	*Monosynaptic cross-modal connections*	**↑** VPM – V1, PF – VPM	—

Summary table displaying the effects of bimodal vs. unimodal stimulation, their underlying mechanisms and anatomical substrate. Black arrows indicate the direction of the observed changes after bimodal stimulation when compared to unimodal stimulation.

Our observed changes in the VPM-S1 circuit after uni- and bimodal stimulation consistently meet the maximum criterion of multisensory integration, i.e. where the response to bimodal visual-tactile stimulation is significantly greater (response enhancement) when compared to unimodal tactile stimulation^44^. In contrast to VPM-S1 where a congruent visual stimulus signifcantly and consistently modulated tactile processing, visual processing in dLGN-V1 was not, or inconsistently, modulated by a congruent tactile stimulus. Thus, given that our analyses fail to consistently meet the maximum criterion of multisensory integration, we have no supporting evidence that dLGN is a multisensory brain region. In contract, our analyses in VPM consistently meet the maximum criterion of multisensory integration demonstrating that this FO thalamic nucleus is a site of visual-tactile convergence. This differential contribution of VPM and dLGN to multisensory integration at thalamic level is in line with our previous observations from the primary sensory cortices, where visual stimuli impact tactile processing stronger than tactile stimuli visual processing^7^. The absence of multisensory integration in dLGN might suggest that neuronal processing in the visual pathway is more eclectic, i.e. the exact configuration of a multisensory stimulus may define which processing strategy is applied^45^. The effects of cross-modal modulation of network activity within VPM are generally weaker from what has been observed at the level of primary sensory cortex^7,8^. However, the impact of visual stimulation on tactile processing in FO thalamus seems to be consistent under different experimental settings, i.e. ranging from simple visual LED light flashes, as used here, to complex movies of natural scenes aiming to recreate natural exploratory behaviour^20^. The differential effects of multisensory integration, which is present in VPM-S1 but absent in dLGN-V1, may have multiple causes. While we cannot fully exclude that the stimulation paradigm optimally revealed visual influence on tactile processing and less the reverse, the temporal alignment of visual and tactile stimuli was controlled before each experiment. Therefore, it is more likely that the cross-modal differences along the visual and tactile tract relate to the fact that multisensory capabilities optimally fit the species-dependent requirements. Taking into account that rats and mice have poor visual acuity, it is very likely that the visual information has a limited behavioural relevance^46,47^, but see also otherwise^48^. Therefore, a visual input might augment the salience of incoming tactile stimuli, yet vice versa it is not the case. The species-specific prevalence of one sense over another has been poorly investigated in rodents. However, it has been shown in visually-dominant species, such as humans and primates, that an eye-centred representation is critical for optimal behavioural outputs, i.e. reaching behaviour, because it specifies the spatial relationship between an object of interest and the required limb movements^49–52^.

Thalamic nuclei mediate multisensory interactions by possibly four distinct mechanisms: (1) individual thalamic nuclei send sensory information to more than one sensory cortex; (2) inputs from multiple senses are integrated at the thalamic level and subsequently sent to sensory cortices, (3) FO thalamic nuclei receive cross-modal inputs from other subcortical areas via other thalamic nuclei; (4) inputs of one sensory area are transferred to thalamus and relayed to another cortical area (cortico-thalamo-cortical route)^53^. Due to the limited temporal resolution of imaging techniques^3,54^, it was previously not possible to distinguish whether thalamic multisensory effects are mediated via feedback connections from cortical areas or whether they are due to bottom-up processing. Our results clearly demonstrate that visual-tactile inputs are integrated already at the level of VPM in FO thalamus, since visual-tactile stimuli caused an enhancement of early evoked potentials and impacted neuronal firing properties of single neurons. By these means, the stimulus impact was augmented, which could decrease behavioural reaction times^55^. Even though the firing probability did not significantly change after bimodal visual-tactile stimulation, the variability of neuronal responses quantified by the Fano factor changed. By these means, multiple sensory inputs can be optimally integrated by weighting their reliability^56^. In line with previous studies^47,57–59^, firing variabilty is a multisensory coding mechanism in VPM, and is independent of significant rate changes. Moreover, the results of anatomical tracing support multisensory integration within thalamo-cortical networks by thalamic innervation of multiple primary sensory cortices. However, this mechanism seems to be restricted to the VPM that sends sparse afferents to V1. The anatomical findings support also cross-modal processing involving other subcortical areas that project to intralaminar thalamus. VPM might receive visual information from SC via strong innervation from PF^60–62^. In contrast, dLGN receives no tactile information, since the upper SC layers that project to dLGN contain mostly visual information-processing neurons.

The convergence of sensory stimuli in VPM might stem from two processing mechanisms. First, the alignment of spontaneous oscillatory activity to a specific phase has been proposed to prime the neural system about forthcoming sensory stimuli, and consequently, to augment their effectiveness^63^. Visual stimuli reset the phase of 1–4 Hz network oscillations in VPM. Thus, a co-occurring tactile stimulus arrives at an optimal phase of ongoing oscillations in VPM. The coincidence of the stimulus with a specific (high or low excitability) phase of network oscillations has been proposed to increase its processing efficiency^64^. Oscillatory phase reset has been previously proposed as an underlying mechanism of cross-modal interactions in primary sensory cortices (S1, V1, auditory cortex)^7,17,32,65^, yet the modulated frequency bands differed between cortical (theta-gamma band) and thalamic areas (delta band). Whether the frequency-specificity in distinct areas reflects the intrinsic patterns of network entrainment, which differ for S1/V1 and FO thalamic nuclei, or selective cross-modal coupling remains to be elucidated. Our data suggest that phase reset of ongoing oscillations is a general mechanism of multisensory interactions used along the sensory tract.

Second, the CFC might act as a mechanism of multisensory integration within thalamo-cortical networks. It has been previously proposed that local and global computations rely on oscillatory entrainment in different frequency bands (i.e. involving a larger number of activated cell populations depending on the length of the oscillatory cycle). The low-frequency phase entrainment combined with phase-amplitude CFC might enhance the communication between distant brain areas during the processing of external sensory stimuli^36^. While the beta-gamma CFC within thalamo-cortical networks was unchanged after unimodal tactile stimulation, it significantly augmented for VPM – S1 (G layers) in the presence of a concurrent visual stimulus. This inter-regional coupling was restricted to the bimodal condition, despite the presence of cortical gamma oscillations induced by uni- and bimodal stimulation. In line with the lack of effects at the level of EPs, similar processes were absent along the visual tract. Bimodal stimulation impacted not only the network oscillations and their temporal relationship, but enhanced also the coupling between spiking activity and neuronal oscillations in VPM-S1. Thus, the alignment of phases in VPM seems to time neuronal firing, thereby augmenting their inter-regional coupling strength (i.e. information exchange) to cortical network oscillations^36^.

One major question that emerges from the present findings is to which extent the cross-modal modulation of network oscillations and neuronal spiking contribute to the efficient processing of multisensory stimuli. To get a first answer, we calculated the directionality of information flow between FO thalamus and primary sensory cortex. Bimodal stimulation augmented the drive from VPM to G layers of S1 and had no effect on the drive from dLGN to the G layers of V1.

The strong cross-modal effects reflecting functional coupling within thalamo-cortical circuits have as anatomical substrate multisensory thalamo-cortical connections. While the phase reset at the level of primary sensory cortices results from mutual projections between S1 and V1^7,13^, similar connectivity between VPM and dLGN is absent. However, VPM also projects to V1. These sparse projections have been identified in gerbils^22^, monkeys^66^ as well as prairie vole^67^. The thalamic neurons projecting to V1 seem to be a distinct population from the majority of those targeting S1, since thalamic neurons projecting to several primary sensory cortices are reportedly quite sparse^22^. These thalamo-cortical connections may facilitate the cross-modal processing along the somatosensory tract. This is in line with the absence of most cross-modal effects along the visual tract, where the corresponding connectivity for dLGN and S1 is lacking.

The present results suggest that visual modulation of VPM facilitates the processing of tactile stimuli even before its convergence within cortico-cortical networks. The findings uncover general principles and mechanisms of multisensory processing, although the rat’s behavioural state during investigation (i.e. sleep-like condition under urethane anaesthesia) and the rather simple stimulation patterns used do not perfectly match the natural multisensory stimulation in behaving animals. Previous studies showed that interactions identified under anesthetised conditions might equally control cross-modal processing in a more natural complex environment^68^. Therefore, we propose that convergence of stimuli in VPM complements the cortico-cortical processing of visual-tactile stimuli and facilitates perceptual discrimination. Performance in whisker-based discrimination tasks has been reported to critically depend on V1 neuronal firing, even in the dark^69^. Moreover, we recently showed that integration of visual and tactile stimuli is mandatory for cross-modal perception^13^. The cross-talk between thalamic and cortical inputs might represent the fastest and most likely, the most efficient way to convey sensory information. The present results demonstrate that cross-modal interactions at thalamic level and within thalamo-cortical networks are powerful instruments for amplifying behaviourally relevant stimuli.

## Methods

### Subjects

The experiments were conducted in compliance with the German laws and the guidelines of the European Community for the use of animals in research and were approved by a local ethical committee (public authority for health and consumer protection of the city of Hamburg, No. 21/10 and 95/15). Brown Norway rats of both sexes (n = 18, weight 32–45 g, age P19–23 at time of surgery/investigation) were obtained from Charles River, housed individually in the animal facility of the University Medical Centre with a 12 h light/12 h dark cycle and fed *ad libitum*. All experiments were conducted during the light phase under sleep-like conditions mimicked by light urethane anaesthesia (0.5 g/kg body weight, i.p., Sigma-Aldrich), thereby avoiding the interference with spontaneous whisking and the impact of alert state that modulates cross-modal processing^7,24^. In contrast to the effects of high urethane doses^70^, the used low concentration did not affect cross-modal responses, as previously shown for the multisensory integration at subcortical level that was similar in anesthetised and awake animals^68^. The network activity and firing was similar during the entire 65 min-long recording and stimulation sessions (Supplementary Fig. S1). The absence of changes in brain state due to wear-off of the anaesthesia in the course of the experiment confirms the lack of urethane confounding effects.

### Surgery

The surgery was performed under ketamine/xylazine anaesthesia (72/9.6 mg/kg body weight, i.p.; Ketavet; Rompun). Body temperature, respiratory rate as well as pain reflexes were monitored during the entire surgery. A circumscribed area of the scalp was removed, the neck muscles were detached from the skull and two metal anchor bars were fixed on the nasal and occipital region with dental cement. Small parts of the skull were removed by drilling holes (0.5 mm in diameter) to expose the dura without causing leakage of cerebrospinal fluid or blood over S1 and V1. The rat’s eyes were covered with ophthalmic ointment (Bepanthen) and the ear canals were filled with silicon adhesive (Kwik-Sil, World Precision Instruments) to block auditory inputs.

### Recording protocols

Extracellular recordings of the LFP and MUA were performed from head-fixed rats using custom made one-shank 32-channel electrodes (0.5–3 MΩ Silicon Michigan probes, Neuronexus Technologies, 100 μm inter-site spacing) that were angularly inserted into S1 barrel field (ventral-medial, 50°, 2.4–2.6 mm posterior and 5.5–5.8 mm lateral to bregma) and V1 (ventral-rostral, 45°, 6.9–7.1 mm posterior and 3.4–3.7 mm lateral to bregma) to a depth of 5.3 mm and 5.5 mm respectively. The placement of recording sites enabled simultaneous recording from the supragranular (S), granular (G) and infragranular (I) layers of S1 and the ventral posteromedial nucleus (VPM) as well as from the S, G and I layers of V1 and the dorsal lateral geniculate nucleus (dLGN). The electrodes were labelled with DiI (1,1′-dioctadecyl-3,3,3′,3′-tetramethyl indocarbocyanine, Invitrogen) for post-mortem reconstruction of their tracks in histological sections (Fig. 1c,d). A silver wire was inserted into the cerebellum and served as ground and reference electrode (Science Products, 99.99% silver, no coating, diameter 200 µm). The body temperature of the animal was kept constant at 37 °C during recording. The electrical activity was recorded at a sampling rate of 32 kHz using a multi-channel extracellular amplifier (no gain, Digital Lynx 10 S, Neuralynx) and the acquisition software Cheetah.

### Sensory stimulation

Unimodal (either light flash or whisker deflection) or bimodal (simultaneous light flash and whisker deflection) stimuli were applied using a custom-made stimulation device as previously described^7,13,16^. Briefly, whiskers were stimulated by deflection through compressed air-controlled roundline cylinders (RT/57110/M/10, Norgren) gated via solenoid valves (VCA, SMCPneumatik), thereby activating subcortical and cortical neurons responding to the principle and adjacent whiskers. The device produced almost silent (6~10 dB, PCE-353), nonelectrical stimulation with precise timing (0.013 ± 0.81 ms) that was constant over all trials/conditions. For full eye field visual stimulation 50 ms-long LED light flashes (300 Lx) were used. For bimodal stimulation whisker deflection and light flashes were applied in the same hemifield. Stimuli were randomly presented across animals in blocks of three different stimulation conditions (unimodal tactile, unimodal visual, bimodal visual-tactile). Each type of stimulus was presented 100 times contralateral to the recording electrodes with an inter-stimulus-interval of 6.5 ± 0.5 s. In line with the *spatial principle of multisensory integration*, visual and tactile stimuli were presented congruently to the same hemifield during bimodal stimulation^44^. In line with the *temporal principle of multisensory integration*, visual-tactile stimuli were simultaneously applied by calculating the time delay of whisker stimulation stemming from the valve-controlled air pressure system and matching the onset of the instantaneous light flash with the onset of the whisker deflection. The non-stimulated eye was covered with aluminium foil patch.

### Retrograde tracing and histology

Retrograde tracing experiments were performed using the retrograde tracers CTB (Alexa Fluor 594 conjugate, ThermoFisher) and hydroxystilbamidine (Fluorogold, Fluorochrome). For CTB injections, seven P14 (dLGN injections: n = 4, VPM injections: n = 3) anesthetised rats (ketamine/xylazine anaesthesia; 72/9.6 mg/kg body weight, intraperitoneal (i.p.) injection; Ketavet; Rompun) were immobilised into a preformed mold fixed into the stereotaxic apparatus. Animals received unilateral injections of CTB conjugated with Alexa Fluor 594 fluorescent dye into VPM (3.0 mm posterior to bregma, 2.4 mm from the midline, to a depth of 4.5 mm) and into dLGN (4.0 mm posterior to bregma, 3.3 mm from the midline, to a depth of 3.7 mm). A working volume of 1% CTB in neutral phosphate buffer was injected via iontophoresis (Midgard Precision Current Source, Stoelting) using positive 4 mA pulses (on/off in 5-s intervals) for a duration of 45 min. Reflux of CTB was avoided by slowly retracting the glass capillary. The surface of the brain was rinsed with sterile water to prevent absorption of, if any, leaked tracer. The surgical opening was sealed with fibrin glue (Surgibond, SMI Sutures) and post-surgery analgesic therapy was given (Meloxicam, 0.1–0.2 mg/kg). After a survival time of 7 days, rats were deeply anesthetised with ketamine/xylazine and perfused transcardially with 4% paraformaldehyde (PFA). The brain was sliced into 50 µm thick coronal slices, air dried, and wide-field images were obtained using an Axioskop 2 Mot microscope (Zeiss) equipped with a fluorescent camera. The sections were examined using the green (460–480 nm) and the red (560–590 nm) excitation filter of the fluorescence microscope. For FG tracing, sections previously used for assessment of cortico-cortical connections^16^ were analysed for thalamo-cortical connectivity using a wide band ultraviolet excitation filter (n = 3).

Fluorescent Nissl staining was performed using the NeuroTrace 500/525 green fluorescent Nissl stain (Invitrogen)^71^. Rehydrated slices were incubated for 20 min with NeuroTrace (dilution 1:100), washed and coverslipped with Fluoromount. The sections were examined using the green (460–480 nm) and the red (535–555 nm) excitation filter of the fluorescence microscope.

### Data analysis

Data were imported and analysed offline using custom-written Matlab scripts (version R2013B, MathWorks). For anti-aliasing, the signal was band-pass filtered (0.1 Hz and 5 kHz) by the Neuralynx recording system. For the LFP, a third-order Butterworth filter was applied before downsampling (by a factor of 10, except for phase-amplitude coupling analysis that was performed on data downsampled by a factor of 80). The position of analysed recording sites over specific cortical layers was confirmed by electrophysiological (i.e. reversal of the EP between S and G layers of the cortex) and histological (i.e. granular cell body layer) landmarks. Further analyses relied on data obtained from one recording site in each cortical layer (S, G, I), the centre of VPM and the core belt of dLGN respectively^72^, where a clear evoked response to tactile or visual stimulation was detected. Only recordings of neuronal activity in rats that showed clear electrophysiological landmarks (sensory evoked potentials, reversal potential in layer 4) and correct electrode positions confirmed *post-mortem* were used for analyses (18 out of 62 used rats).

#### Calculation of evoked potentials

Continuous recordings in VPM and dLGN were epoched offline and 1 s-long time windows (300 ms pre-stimulus and 700 ms post-stimulus) corresponding to each stimulation condition (unimodal visual, unimodal tactile, bimodal visual-tactile) were averaged for each animal. The EP peaks were detected as local LFP maxima/minima within a sliding time window of 100 ms. The amplitude peaks were defined as positive (P) or negative (N) based on surface polarity. The EP latency was defined as the time point of positive or negative amplitude peak after stimulation. The mean amplitude and mean peak latency from stimulation onset were averaged across trials for each recording site and all animals, and tested for significant differences between unimodal and bimodal stimulation.

#### Spectral analysis of baseline activity

To identify the main frequency bands of baseline (i.e. stimulus unrelated) activity, power spectral density estimates of 30 min of neural activity (15 min preceding and 15 min following the sensory stimulation protocol) were calculated. For this, we used the MATLAB function *pwelch* with 3 s-long segments having an overlap of 50%. Each value of the obtained power spectral density estimate was multiplied by the frequency value to the power of 1.5.

#### Spectral analysis of induced activity

For each stimulation trial, continuous wavelet coefficients C were calculated for time windows of 3000 ms (1500 ms pre-stimulus and 1500 ms post-stimulus) at frequency scale *a* and position *b* by the following formula

$${{\rm{C}}}_{{\rm{a}},{\rm{b}}}={\int }_{{\rm{R}}}^{\,}{\rm{\Phi }}({\rm{t}})\frac{1}{\sqrt{{\rm{a}}}}\overline{{\rm{\Psi }}(\frac{{\rm{t}}-{\rm{b}}}{{\rm{a}}})}{\rm{dt}}$$

where Ψ is a Morlet wavelet. Wavelets were corrected for pink noise by normalisation to the coefficients of baseline activity (500 to 1000 ms before stimulus) at every frequency. Baseline normalised wavelets were averaged across all rats. To detect a significant modulation of induced activity, the power modulation was averaged across trials for each animal and transformed into a z-score by

$${Z}_{tf}=\frac{activit{y}_{tf}-\overline{baselin{e}_{f}}}{\sqrt{{n}^{-1}{\sum }_{i=1}^{n}{(baselin{e}_{if}-\overline{baselin{e}_{f}})}^{2}}}$$

where n is the number of time points in the baseline period, whereas *t* and *f* correspond to time and frequency points, respectively. Z-score normalised wavelets were averaged across animals. Values higher than 2.576 or lower than −2.576 (99% confidence interval, p < 0.001) reflect significant differences in power before and after stimulation. Based on the statistical values, time windows with stimulus-modulated frequency were detected. The mean coefficients of the baseline-normalised wavelets within these windows after unimodal versus bimodal stimulation were tested for significant differences across animals. The amplitude of induced oscillations in different frequency bands was calculated for individual trials (300 ms before stimulus and 700 ms after stimulus) using the Hilbert transform and averaged for all trials. Mean amplitude of oscillations during time windows pre- (200–300 ms) and post-stimulus (50–150 ms; 300–400 ms) were tested for significant differences. The time window of 0–50 ms after stimulation was excluded to avoid an artificial increase in oscillatory power due to the sensory evoked potential.

#### Phase analysis

The phase distribution across trials was estimated by calculating the mean length of the resulting vector. For this, single trials of LFPs during a time window of 1 s (500 ms pre-stimulus and 500 ms post-stimulus) were filtered using a third-order Butterworth filter in three different frequency ranges identified by power spectral density analysis of baseline activity (1–4 Hz, 4–12 Hz, 12–45 Hz) (Supplementary Fig. S1). The phase of oscillatory activity was extracted using the Hilbert transform and single trial event-related phase values were analysed by circular statistical methods^73^. Due to zero-phase digital filtering, the phase was not distorted but the time resolution of phase distribution was poor. The mean resultant vector (MRV) length was calculated at each frequency and time point, and normalised to baseline (200–300 ms before stimulus). The vector length indicates the phase consistency over trials and ranged between 0 (no phase synchrony) and 1 (max. phase synchrony). Significant phase synchrony after stimulation was assessed by the 99% confidence interval z-score values. The confidence interval values of the population mean were calculated at each time point after stimulation based on the formula

$$\bar{{\rm{x}}}\pm Z\ast \frac{\sigma }{\sqrt{n}}$$

where Z is the z-score representing the 0.001 alpha level, σ the standard deviation of the phase distribution and n the number of animals. The interval value obtained from the formula was added or subtracted from the population mean to find the upper and the lower end of the confidence range.

Event-related phase synchrony may result from (i) the phase locking of evoked responses to the sensory stimulus, (ii) the alignment of oscillatory phases to the sensory stimulus (i.e. true synchrony), or (iii) a combination of both. Evoked responses are accompanied by a pre- to post-stimulus power increase in the single-trial responses that is not present after the alignment of oscillatory phases to the sensory stimulus^33,34^. To distinguish the true phase synchrony, we compared the MRV values to the pre- to post-stimulus power ratio ([0–250 ms post-stimulus]/[200–300 ms pre-stimulus]) for each trial. A power-ratio equal to one and an MRV larger than zero correspond to the true reset of oscillatory phase.

#### Phase-amplitude cross-frequency coupling

As previously reported^38,74,75^, wavelets were used to generate the analytic phase of the low frequency signal ${{\o}}_{fph}(t)$ and the analytic amplitude of the high frequency signal ${A}_{fam}(t)$. Subsequently, a composite signal was calculated as

$${Z}_{fph,fam}(t)={A}_{fam}(t)\ast \exp (i\ast {{\o}}_{fph}(t)).$$

The modulation index (MI), which measures the dependence of the amplitude of the high-frequency signal on the phase of the low-frequency signal, was calculated as

$$M{I}_{raw}=abs(mean({Z}_{fph,fam}(t)))$$

and normalised using surrogate data to Z-score values, which were generated by introducing a time lag τ between ${{\o}}_{fph}(t)$ and ${A}_{fam}(t)$ by

$${Z}_{surr}(t,{\rm{\tau }})={A}_{fam}(t+{\rm{\tau }})\ast \exp ({\rm{i}}\ast {{\o}}_{fph}(t))$$

The resulting $M{I}_{Norm}$ was defined as

$$M{I}_{Norm}=(M{I}_{raw-}\mu )/\sigma ,$$

where *μ* was the mean and $\sigma$ was the standard deviation of the surrogate data.

The instantaneous phase and amplitude were extracted from the 1–20 Hz and the 30–100 Hz filtered signal in steps of 1 Hz and 2 Hz, respectively. Pre- (100–600 ms) and post-stimulation (100–600 ms) MI values were calculated and normalized to a z-score using the mean and standard deviation (SD) of a distribution of phase-amplitude CFC values obtained from 50 surrogates. These surrogates were created by combining the instantaneous phase and amplitude values for variable time lags. The relative change in MI values was obtained by subtracting the pre- from the post-stimulus values.

#### Generalised partial directed coherence

Generalised partial directed coherence (gPDC) was used to measure the strength of directional coupling between thalamic nuclei and primary sensory cortices after uni- and bimodal stimulation. The method relies on vector autoregressive (VAR) modelling^41^.

Briefly, for signal ${X}_{t}$ and ${Y}_{t}$, the VAR model with order p was determined as

$$[\begin{array}{c}{X}_{t}\\ {Y}_{t}\end{array}]=\sum _{r=1}^{p}[\begin{array}{cc}{a}_{r}^{11} & {a}_{r}^{12}\\ {a}_{r}^{21} & {a}_{r}^{22}\end{array}][\begin{array}{c}{X}_{t-r}\\ {Y}_{t-r}\end{array}][\begin{array}{c}{{\rm{\varepsilon }}}_{t}^{X}\\ {\varepsilon }_{t}^{Y}\end{array}]$$

and the Fourier transformation was applied to the VAR coefficients

$$A(f)=\sum _{r-1}^{p}[\begin{array}{cc}{a}_{r}^{11} & {a}_{r}^{12}\\ {a}_{r}^{21} & {a}_{r}^{22}\end{array}]\ast \exp (\,-\,i2\,{\rm{\pi }}f).$$

The difference between the identity matrix and $A(f)$ was defined as $\bar{A}(f)$

$$\bar{A}(f)=[\begin{array}{cc}1 & 0\\ 0 & 1\end{array}]-\sum _{r-1}^{p}[\begin{array}{cc}{a}_{r}^{11} & {a}_{r}^{12}\\ {a}_{r}^{21} & {a}_{r}^{22}\end{array}]\ast \exp (-i2\,{\rm{\pi }}{\rm{f}})=[\begin{array}{cc}{a}_{11}(f) & {a}_{12}(f)\\ {a}_{21}(f) & {a}_{22}(f)\end{array}]$$

The gPDC from area Y to area X was calculated as

$$gPD{C}_{Y\to X}({\rm{f}})=\frac{\frac{1}{{\sigma }_{2}}{a}_{21}({\rm{f}})}{{a}_{11}(f)\frac{1}{{\sigma }_{1}\,}+{a}_{22}(f)\frac{1}{{\sigma }_{2}}},$$

where ${\sigma }_{1}$ and ${\sigma }_{2}$ represented the standard deviation of the model residuals.

The gPDC was calculated for 100–600 ms both post- and pre-stimulus. The post-stimulus values were divided by the pre-stimulus values to obtain the relative partial directed coherence change after stimulation.

#### Spike sorting and cluster analysis

The raw signal was high-pass filtered (>400 Hz). The threshold for detecting MUA was set individually at 25–30 µV. The stored signals were semi-automatically sorted offline depending on waveform shape using spike sorting software (Plexon). A group of similar waveforms was considered as being generated from a single neuron if it defined a discrete cluster in a 2D/3D space and exhibited a clear refractory period (>1 ms) in the interspike interval histogram. The quality of separation between identified clusters was assessed by four different statistical measurements: the classical parametric F statistic of multivariate analysis of variance (MANOVA), the J3 and PseudoF (Psf) statistics and the Davies-Bouldin validity index (DB)^76,77^. The values of statistical testing in VPM ranged between 6 × 10^−36^ and 0.02 for MANOVA, 1.14 and 11.08 for J3, 5761 and 83286 for PsF, and 0.14 and 0.68 for DB and in dLGN they ranged between 7 × 10^−15^ and 0.05 for MANOVA, 1.50 and 6.40 for J3, 585 and 477793 for PsF, and 0.07 and 0.71 for DB. A total number of 126 units were clustered in VPM and 66 in dLGN. Approximately one to four units per recording site could be extracted in VPM and dLGN.

#### Firing probability

Firing probability of single-unit activity (SUA) was calculated 100 ms before and 500 ms after stimulus (1 ms bin size) and averaged across trials. Units were considered as being responsive if the stimulus-induced firing probability (5–25 ms post-stimulus) was larger or smaller than five times the baseline firing probability (5–55 ms pre-stimulus). Based on this criterion, only the neurons that showed a response to unimodal sensory stimulation were considered for the analysis of cross-modal rate changes. To quantify the enhanced responses of single units after uni- vs. bimodal stimulation, we computed the FF, which measures the response variability of single units to sensory stimulation^58,59^. The FF was calculated as

$$FF=\frac{{{\rm{\sigma }}}^{2}}{{\rm{\mu }}},$$

where the variance (σ^2^) and mean (μ) of spike counts was computed across trials and averaged over 5–25 ms after stimulation in VPM and over 25–55 ms after stimulation in dLGN. Stimulation-related $F{F}_{stim}$ was then normalised to the baseline $F{F}_{baseline}$ $(F{F}_{stim}/F{F}_{baseline})$, which was calculated as the mean value of FF within 5–100 ms before stimulation (50% overlap with 20 ms and 30 ms calculation window in VPM and dLGN, respectively).

#### Spike-LFP coupling

Thalamo-thalamic and thalamo-cortical phase locking of spiking of clustered units to network oscillations was assessed using a previously described algorithm^71,78^. For this, the raw LFP signal was bandpass filtered (1–4 Hz, 4–12 Hz, and 12–45 Hz) using a third-order Butterworth filter preserving phase information. Subsequently, a Hilbert transform was applied to the filtered signal. If the firing of a neuron was modulated by oscillations within a specific frequency band, then its phase over the oscillatory cycle was not uniformly distributed. Phases of zero referred to the peak and a phase of π/-π referred to the trough of the cycle. The coupling between spikes and network oscillations was tested for significance using the Rayleigh test for non-uniformity. The spike trains were converted into a sequence of unit length vectors oriented by the phase of their corresponding spikes. The value of Rayleigh’s *Z* statistic indicates strength of phase coupling (or degree of non-uniformity) between unit events and field potential and was computed as

$$Z=n{R}^{2}$$

where R denotes the MRV length of the given phase series. The probability that the null hypothesis of sample uniformity holds is given by

$$P={e}^{-z}[1+\frac{2Z-{Z}^{2}}{4n}-\frac{24Z-132{Z}^{2}+76{Z}^{3}-9{Z}^{4}}{288{n}^{2}}]$$

For *n* 50, ${\rm{P}}={{\rm{e}}}^{-{\rm{Z}}}$ approximation is adequate^79^. Only neurons that showed a significant degree of phase locking were considered for analyses of temporal changes. Their MRV length (locking strength) as well as their mean direction (preferred phase of locking) were calculated.

### Statistical analysis

Unimodal tactile, unimodal visual and bimodal visual-tactile stimulations were presented to all animals in a repeated measurement design. The order of tactile, visual and bimodal stimulation blocks (50 trials each) were randomised and repeated twice (total of 100 trials of each stimulation type). Statistical analyses were performed using Matlab (version R2013B) or SPSS Statistics (version 22.0, IBM). Gaussian distribution of the data was assessed using the Kolmogorov-Smirnov test. Normally distributed data were tested with the paired t-test. Data that did not follow a Gaussian distribution were tested with the Wilcoxon signed-rank test for paired data. Bonferroni-corrections were applied for multiple comparisons. Count data were analysed with the two proportion *z*-test. Non-uniformity of circular data was assessed using the Rayleigh test. Significant differences in the preferred phase of neuronal firing to oscillatory activity were assessed using the nonparametric *circ_cm* test of the Matlab circular statistics toolbox^73^. Two-tailed statistical tests were used if not stated otherwise. Data are shown as mean ± SEM.

## Electronic supplementary material

Supplementary Material

## Data Availability

All data needed to evaluate the conclusions in the paper are present in the paper and/or the supplementary material. Data can be made available by the authors upon request.
