# Supplementary material for: Multisensory integration in rodent tactile but not visual thalamus
Source: Sci Rep. 2018 Oct 24;8:15684. doi: 10.1038/s41598-018-33815-y (PMC6200796; doi:10.1038/s41598-018-33815-y)
Supplement: Supplementary file 1 — Supplementary Material [file 41598_2018_33815_MOESM1_ESM.pdf]

Multisensory integration in rodent tactile but not visual thalamus

Malte Bieler, Xiaxia Xu, Annette Marquardt & Ileana L. Hanganu-Opatz

Supplementary Material

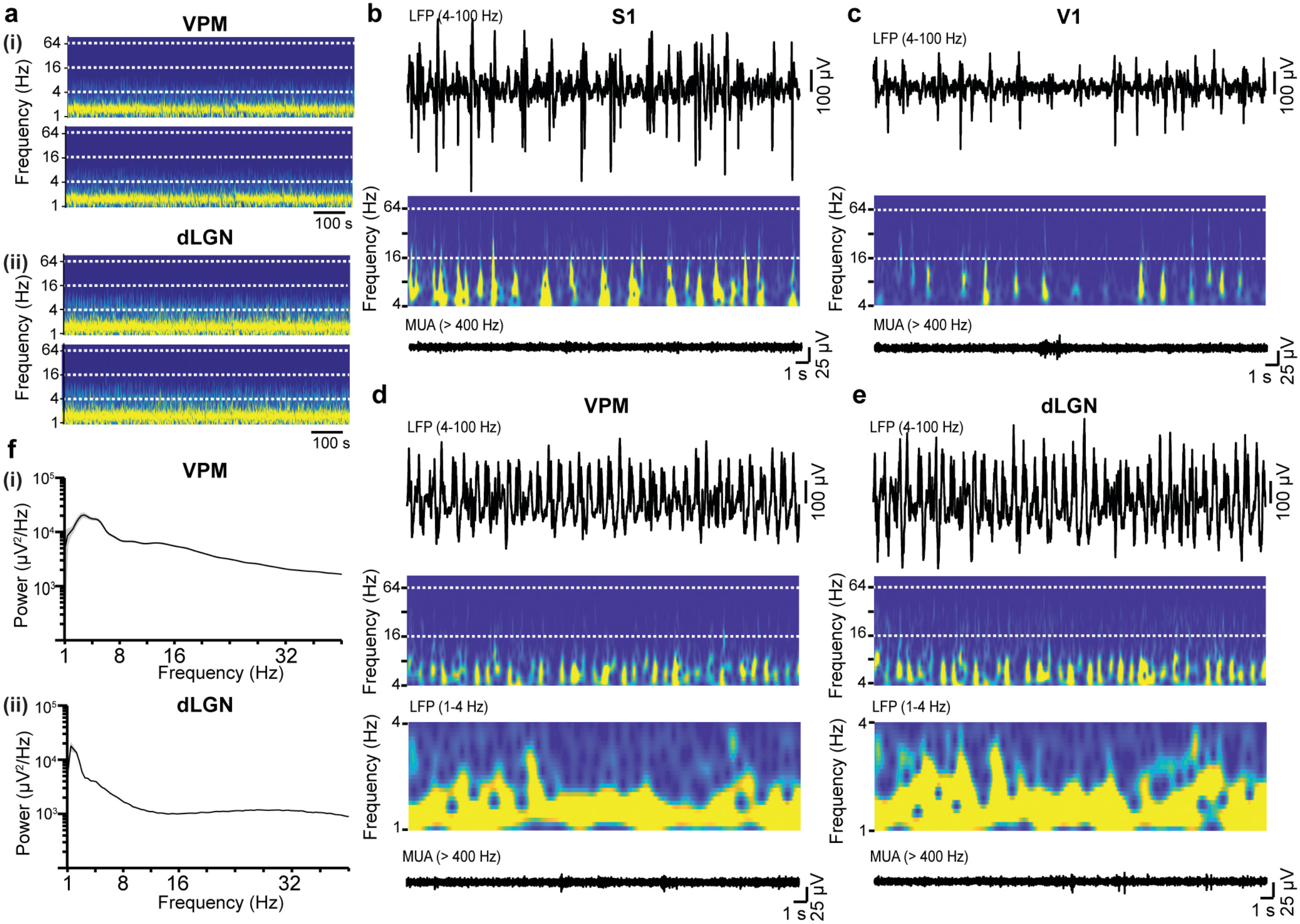

a
VPM
(i)
Frequency (Hz)
100 s
dLGN
(ii)
Frequency (Hz)
100 s
b
LFP (4-100 Hz)
S1
100 µV
Frequency (Hz)
MUA (> 400 Hz)
1 s
25 µV
c
V1
LFP (4-100 Hz)
100 µV
Frequency (Hz)
MUA (> 400 Hz)
1 s
25 µV
d
VPM
LFP (4-100 Hz)
100 µV
Frequency (Hz)
LFP (1-4 Hz)
Frequency (Hz)
MUA (> 400 Hz)
1 s
25 µV
e
dLGN
LFP (4-100 Hz)
100 µV
Frequency (Hz)
LFP (1-4 Hz)
Frequency (Hz)
MUA (> 400 Hz)
1 s
25 µV
f
VPM
(i)
Power (µV²/Hz)
Frequency (Hz)
dLGN
(ii)
Power (µV²/Hz)
Frequency (Hz)

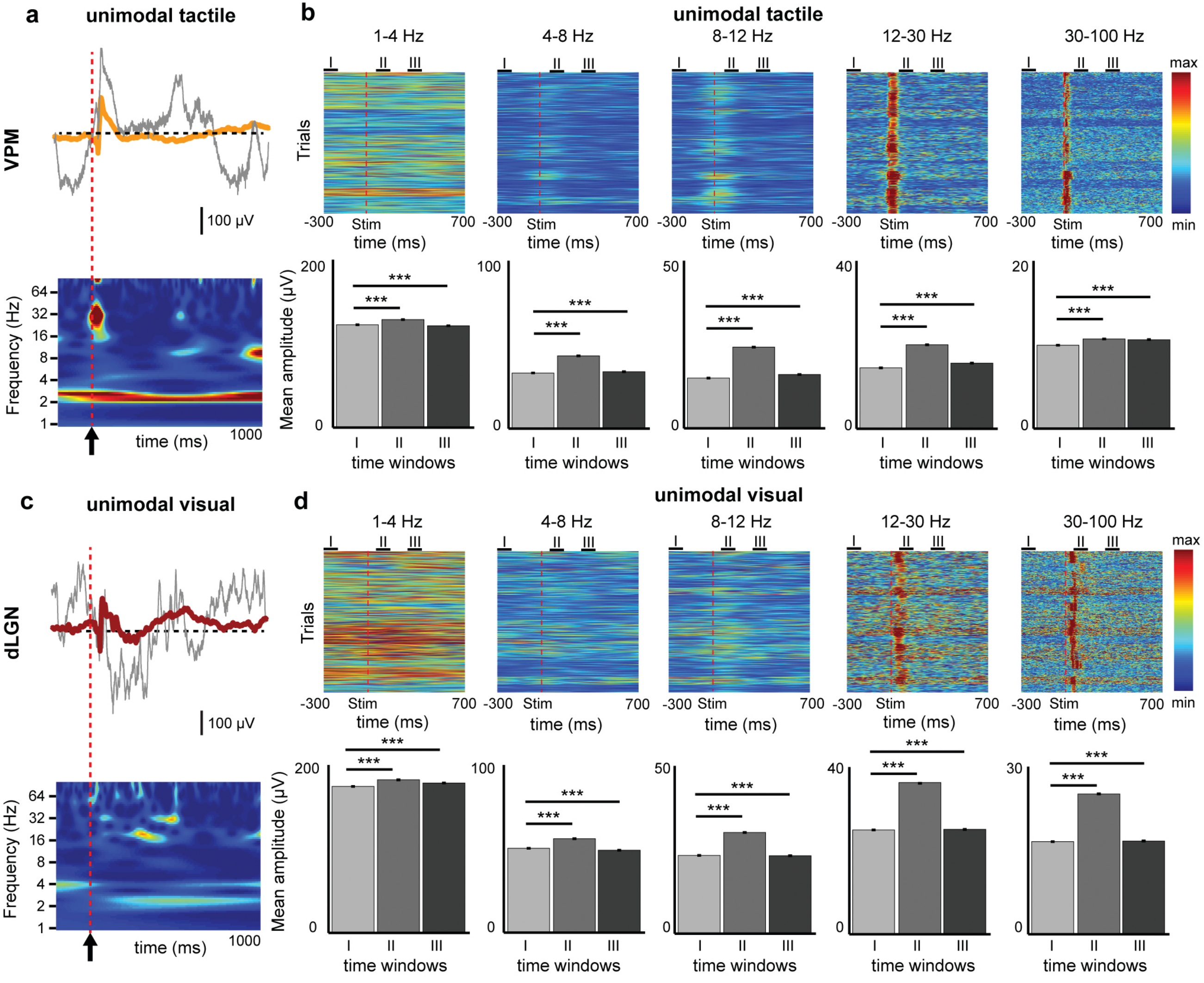

a
unimodal tactile
VPM
100 µV
Frequency (Hz)
64
32
16
8
4
2
1
time (ms)
1000
b
unimodal tactile
1-4 Hz
4-8 Hz
8-12 Hz
12-30 Hz
30-100 Hz
I
II
III
Trials
max
min
-300
Stim
700
time (ms)
Mean amplitude (µV)
200
100
50
40
20
0
***
time windows
c
unimodal visual
dLGN
100 µV
d
unimodal visual
30

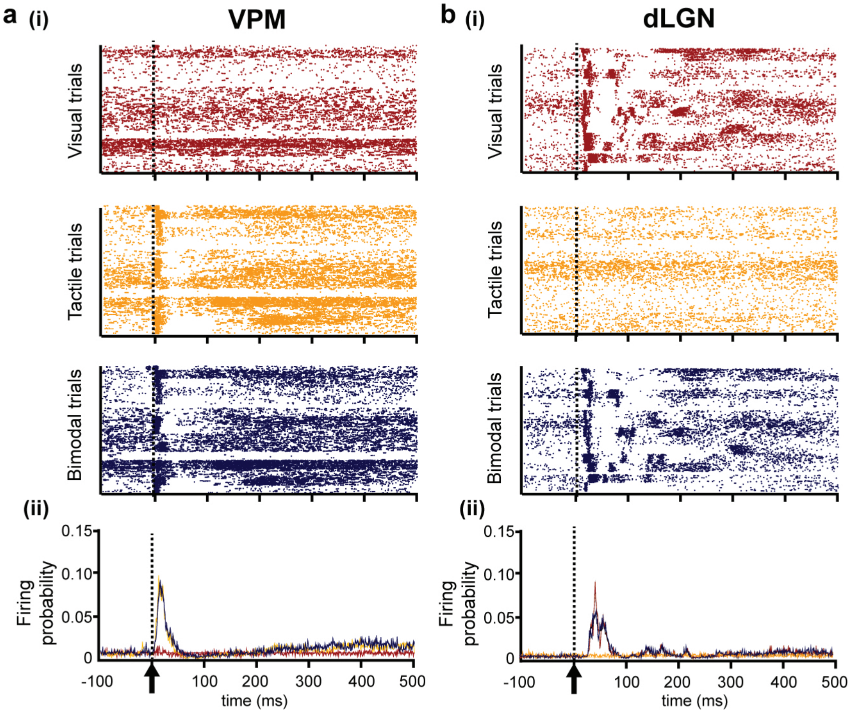

a
(i)
VPM
Visual trials
Tactile trials
Bimodal trials
(ii)
Firing probability
0.15
0.10
0.05
0
-100
100
200
300
400
500
time (ms)
b
(i)
dLGN
Visual trials
Tactile trials
Bimodal trials
(ii)
Firing probability
0.15
0.10
0.05
0
-100
100
200
300
400
500
time (ms)

Supplementary Table S1. Amplitude (μV) of stimulus-induced oscillations in FO thalamus after unimodal stimulation.

	1-4 Hz			4-8 Hz			8-12 Hz			12-30 Hz			30-100 Hz		
	I	*II*	*III*	*I*	*II*	*III*	*I*	*II*	*III*	*I*	*II*	*III*	*I*	*II*	*III*
**VPM**	121.76 ± 0.08	128.65 ± 0.10	120.55 ± 0.10	32.73 ± 0.03	42.97 ± 0.04	33.36 ± 0.02	14.93 ± 0.02	23.95 ± 0.02	15.89 ± 0.02	14.35 ± 0.02	19.97 ± 0.02	15.48 ± 0.01	9.99 ± 0.02	10.78 ± 0.01	10.61 ± 0.01
**dLGN**	177.2 ± 0.12	185.4 ± 0.15	182.2 ± 0.15	50.32 ± 0.05	56.22 ± 0.05	49.13 ± 0.04	23.27 ± 0.03	30.2 ± 0.03	23.11 ± 0.02	25.48 ± 0.03	37.29 ± 0.04	25.79 ± 0.03	16.52 ± 0.02	25.06 ± 0.03	16.76 ± 0.02

Supplementary Table S2. Baseline power (10^4 μv^2) in FO thalamus and cortical layer IV

	**1-4 Hz**	**4-12 Hz**	**12-30 Hz**	**30-100 Hz**
S1_IV	18.84 ± 7.63	1.78 ± 0.53	0.36 ± 0.09	0.10 ± 0.03
VPM	6.71 ± 1.16	0. 70 ± 0.12	0.11 ± 0.03	0.04 ± 0.01
V1_IV	2.54 ± 0.46	0.95 ± 0.19	0.24 ± 0.05	0.05 ± 0.01
dLGN	13.70 ± 3.24	1.85 ± 0.45	0.42 ± 0.14	0.16 ± 0.06

Legends

Supplementary Figure S1. Patterns of spontaneous electrical activity in primary sensory cortices and corresponding first-order thalamic nuclei. **(a)** (i) Wavelet spectrum of oscillatory activity (1-100 Hz) recorded before (top) and after (bottom) the 65 min-long stimulation session in the VPM of a P21 rat**. (ii)** Same as (i) for dLGN. **(b)** Extracellular LFP recording of spontaneous oscillatory activity in the S1 of a P21 rat and the corresponding MUA after 400 Hz high-pass filtering displayed together with the color-coded wavelet spectrum (signal filtered at 4-100 Hz) at identical time scale. **(c)** Same as (b) for V1. **(d)** Same as (b) for VPM. **(e)** Same as (b) for dLGN. **(f)** Normalised power spectrum of spontaneous oscillatory activity recorded in VPM (i) and dLGN (ii).

Supplementary Figure S2. Unimodal induced responses in the first-order thalamic nuclei. (a) Single (grey) and averaged (orange) responses to tactile stimulation (red dotted line and black arrow) in VPM (top) accompanied by the corresponding baseline normalised Morlet wavelet spectra (bottom) of a single whisker stimulation trial. **(b)** Top, temporal profile of tactile-induced network oscillations in different frequency bands from all investigated rats. The color-coded plots were used to identify the time windows (I, II, III) during which the amplitude of induced oscillations is modified in all trials. Bottom, bar diagrams displaying the mean amplitude of induced oscillations during the identified time windows. **(c)** Same as (a) for dLGN. **(d)** Same as (c) for dLGN. (*$p<0.05$, **$p<0.01$, ***$p<0.001$; Kruskal Wallis, Bonferroni-Holm corrected).

Supplementary Figure S3. Neuronal firing in first-order thalamus after visual, tactile and visual-tactile stimulation. (a) (i) Rasterplot depicting the firing of single neurons in VPM after visual (top, red), tactile (orange, middle) and bimodal visual-tactile (blue, bottom) stimulation trials. Black arrow and dotted black line mark the stimulation. (ii) Line plot

showing the firing probability of VPM neurons after visual (red), tactile (orange) and visual-tactile (blue) stimulation. **(b)** Same as (a) for dLGN.

Supplementary Table S1. Amplitude of stimulus-induced oscillations in FO thalamus after unimodal stimulation. Amplitude values (μV) are mean ± SEM for stimulus induced oscillations in defined frequency bands in FO thalamic nuclei. I, II, and III correspond to the following investigated time windows: 200-300 pre-stimulus, and 50-150 ms and 300-400 ms post-stimulus.

Supplementary Table S2. Baseline power (10^4 μv^2) in FO thalamus and cortical layer IV. Power values of oscillatory activity in FO thalamic nuclei and primary sensory cortices (n=8) displayed as mean ± SEM.
